# Prediction of restenosis based on hemodynamical markers in revascularized femoro-popliteal arteries during leg flexion

**DOI:** 10.1007/s10237-019-01183-9

**Published:** 2019-06-13

**Authors:** Can Gökgöl, Nicolas Diehm, Lorenz Räber, Philippe Büchler

**Affiliations:** 1 ARTORG Center for Biomedical Engineering Research, University of Bern, Bern, Switzerland; 2 Clinical and Interventional Angiology, Vascular Institute Central Switzerland, Aarau, Switzerland; 3 Department of Cardiology, Inselspital, Bern University Hospital, University of Bern, Bern, Switzerland

**Keywords:** Femoro-popliteal (FP) arteries, Patient-specific, Endovascular therapy, Leg flexion, Computational fluid dynamics (CFD), 2D/3D reconstruction

## Abstract

Endovascular therapy in patients suffering from peripheral arterial disease shows high rates of restenosis. The poor clinical outcomes are commonly explained by the demanding mechanical environment due to leg movements, but the mechanisms responsible for restenosis remain unknown. In this study, we hypothesized that restenosis following revascularization is associated with hemodynamical markers derived from blood flow during leg flexion. Therefore, we performed personalized computational fluid dynamics (CFD) analyses of 20 patients, who underwent routine endovascular femoro-popliteal interventions. The CFD analyses were conducted using 3D models of the arterial geometry in straight and flexed positions, which were reconstructed from 2D angiographic images. Based on restenosis rates reported at 6-month follow-up, logistic regression analyses were performed to predict restenosis from hemodynamical parameters. Results showed that severe arterial deformations, such as kinking, induced by leg flexion in stented arteries led to adverse hemodynamic conditions that may trigger restenosis. A logistic regression analysis based solely on hemodynamical markers had an accuracy of 75%, which showed that flow parameters are sufficient to predict restenosis (*p* = 0.031). However, better predictions were achieved by adding the treatment method in the model. This suggests that a more accurate image acquisition technique is required to capture the localized modifications of blood flow following intervention, especially around the stented artery. This approach, based on the immediate postoperative configuration of the artery, has the potential to identify patients at increased risk of restenosis. Based on this information, clinicians could take preventive measures and more closely follow these patients to avoid complications.

**Electronic supplementary material:**

The online version of this article (10.1007/s10237-019-01183-9) contains supplementary material, which is available to authorized users.

## Introduction

Obstructions of the femoro-popliteal (FP) arteries in the lower limbs of the body—clinically called peripheral arterial disease (PAD)—affect about 20% of the population over 70 years of age (Norgren et al. 2007). Moreover, the worldwide rate of PAD incidents is constantly increasing, suggesting that the disease will continue to be a significant burden on the healthcare systems (Fowkes et al. 2013).

Nowadays, PAD is mainly treated with endovascular therapy, which can be performed with different methods such as percutaneous transluminal angioplasty (PTA), mechanical or laser atherectomy, or stent implantation (Norgren et al. 2007; Shammas 2017). With the invention of drug-eluting technology, the field has actively favored the strategy of “leaving nothing behind” (Bosiers 2013; Casserly 2017). As a result, performing PTA with drug-coated balloons is progressively replacing the use of stents as the predominant treatment method for PAD, with stents being implanted only if balloon angioplasty produces sub-optimal results (Casserly 2017). However, even with this change in paradigm, the rates of primary patency and freedom from target lesion revascularization (TLR) have not shown significant improvements. This observation is confirmed by long-term outcomes from two recent controlled clinical studies; the 2-year results of the Lutonix SFA Registry report a primary patency of 76% and a TLR rate of 11%, which are similar to the 1-year results observed in the DEFINITIVE AR study (Thieme et al. 2017; Zeller et al. 2017). Furthermore, these values are more likely to increase in non-controlled clinical trials as real-world outcomes tend to show higher restenosis rates (Iida et al. 2015).

The main reason behind these adverse outcomes is hypothesized to be associated with the repeated mechanical loads imposed on these arteries during flexion of the leg (Smouse and Nikanorov 2005; Cheng et al. 2010; Diehm et al. 2011). However, until recently, the deformation behavior of the FP arterial tract was only poorly understood. Although several studies have shown that this arterial segment is exposed to significant levels of axial deformation, twisting, and bending due to varying degrees of hip/knee flexion, there was very limited information on its post-treatment behavior (Choi et al. 2009; Klein et al. 2009; Gökgöl et al. 2013; MacTaggart et al. 2014). As a result, it was not possible to identify a relationship between the deformations altered by endovascular therapy and the occurrence of restenosis observed on patients. Recent studies have tried to establish this link by examining the post-treatment arterial deformations of PAD patients (Ganguly et al. 2011; Nikanorov et al. 2013; Gökgöl et al. 2017; Schumann et al. 2017; MacTaggart et al. 2018). Among these, the publications of Gökgöl et al. characterized the changes in FP artery deformations due to endovascular treatment (pre- vs. post-treatment) and between different treatment methods (PTA vs. nitinol stent implantation; Gökgöl et al. 2017; Schumann et al. 2017). More significantly, they showed a correlation between arterial kinking due to leg flexion and restenosis observed at 6-month follow-up, which further supported the hypothesis that severe arterial deformations are the main driving force behind adverse clinical outcomes (Gökgöl et al. 2017). Nevertheless, the underlying mechanisms responsible for restenosis remain incompletely understood and require further analysis.

The deformations caused by leg flexion not only affect the mechanical behaviors of the artery and nitinol stent, but also the characteristics of blood flow (Ní Ghriallais and Bruzzi 2014). The motion-induced changes in curvature and radial diameter (i.e., pinching) of the artery are expected to strongly impact the flow profile and, consequently, increase the areas affected by atheroprone flow conditions, such as low/high wall shear stress (WSS) or high oscillatory shear index (OSI). The changes in these flow parameters due to stent implantation in both coronary and peripheral arteries have been the subject of numerical investigations through computational fluid dynamics (CFD) analyses (LaDisa et al. 2005; Williams et al. 2010; Rikhtegar et al. 2013; Morlacchi et al. 2013; Chiastra et al. 2015; Gökgöl et al. 2015). In contrast, there are only a limited number of studies that analyzed the influence of arterial geometry on FP artery hemodynamics and even less that concentrated on the alterations in the flow dynamics due to flexion-induced geometrical changes in the arterial lumen (Wood et al. 2006; Kim et al. 2008; Xu et al. 2016; Desyatova et al. 2017). The majority of these studies performed patient-specific flow simulations with artery models in supine position and showed that the native curvature and tortuosity of healthy and diseased FP arteries have a significant role in the changes in the natural hemodynamic factors (Wood et al. 2006; Kim et al. 2008). However, to the best of our knowledge, there is only one study that investigated the hemodynamics of FP arteries with the leg in a flexed position (Desyatova et al. 2017). CFD analyses were used to estimate the effects of age on the WSS parameters in models that incorporated population-specific diameter information. However, their models did not include patient-specific characteristics of the arterial lumen, such as the local variations in the arterial cross section. In addition, the study only considered a single-leg position (i.e., gardening position) and, as such, the results do not represent the effects of leg flexion. Moreover, no study evaluated the relationship between the changes in the hemodynamical behaviors induced by leg flexion and clinical outcomes.

This study aims to quantify the flow behavior of the FP arteries following endovascular revascularization on a series of 20 patients and to compare the results of the numerical analyses with instances of restenosis reported at 6-month follow-up. Our hypothesis is that patient-specific CFD simulations can provide hemodynamical markers that are able to predict the risk of restenosis.

## Methods

The angiographic data of 20 patients that had been acquired as part of a clinical investigation were used for this study, which was approved by the local ethics committee (EKNZ 2014-119). Each patient signed a consent form for inclusion in the study. Detailed information on the patient characteristics and treatment procedures can be found in Table 1, as well as in our previous publications (Gökgöl et al. 2017; Schumann et al. 2017). In summary, the patients (mean age 73 ± 9; 11 females; stage of PAD: IIB) were scheduled for routine endovascular treatment. Prior to the start of the procedure, a calibration phantom was attached to the patients’ diseased leg. Based on the lesions and the success of the initial PTA, ten patients underwent only balloon angioplasty, whereas the rest were implanted with nitinol stents. Based on X-ray images acquired immediately after the treatment, seven patients (all stented) exhibited arterial kinking when the leg was flexed. All patients underwent post-interventional duplex sonography scanning, and hemodynamically relevant residual stenosis was thereby ruled out in all patients. At a routine 6-month follow-up, seven patients (five stented) had shown restenosis, which was diagnosed based on duplex sonography and defined by a peak systolic velocity ratio greater than 2.4 at the target lesion (Ranke et al. 1992). These patients did not undergo additional treatment within the frame of this study and, as such, no angiographic follow-up was performed.

**Table 1:** Lesion characteristics, treatment details, presence of arterial kinking with leg flexion, and clinical outcome at 6-month follow-up

Patient	Lesion location	Lesion length (mm)	Level of calcification	Brand of the stent/balloon; diameter (mm) × length (mm)	Kinking/restenosis
1	Distal SFA/popliteal	180	Moderate	Pulsar 18^a^; 6 × 200	+/+
2	CFA/distal SFA	350	Severe	2× Protégé Everflex^b^; 6 × 200	+/+
3	Mid-/distal SFA	350	Severe	Pulsar 18; 5 × 200	+/+
4	Distal SFA/popliteal	180	Moderate	Pulsar 18; 6 × 200	+/+
5	Popliteal	70	Moderate	Pulsar 18; 5 × 80	+/+
6	Distal SFA	100	Moderate	Zilver PTX^c^; 6 × 120	+/−
7	Mid-/distal SFA	100	Moderate	Zilver PTX; 6 × 120	+/−
8	Proximal/distal SFA	400	Severe	2 × Protégé Everflex; 6 × 200	−/−
9	Proximal/distal SFA	350	Severe	2 × Pulsar 18; 5 × 200	−/−
10	Mid-SFA	80	Severe	Protégé Everflex; 5 × 100	−/−
11	Popliteal	80	Moderate	3 × PTA; 4 × 40	−/+
12	Mid-/distal SFA	10	Moderate	PTA; 6 × 20	−/+
13	Distal SFA	50	Moderate	PTA; 4 × 40	−/−
14	Distal SFA	40	Moderate	PTA; 4 × 40	−/−
15	Distal SFA	10	Moderate	PTA; 4 × 20	−/−
16	Mid-SFA	50	Moderate	PTA; 5 × 40	−/−
17	Mid-/distal SFA	50	Moderate	PTA; 5 × 40	−/−
18	Mid-/distal SFA	40	Moderate	PTA; 4 × 60	−/−
19	Distal SFA/popliteal	80	Moderate	2 × PTA; 4 × 40	−/−
20	Mid-/distal SFA	100	Moderate	3 × PTA; 5 × 40	−/−

*CFA* common femoral artery, *SFA* superficial femoral artery, *PTA* percutaneous transluminal angioplasty; *+/−* represents true/false conditions

^a^Biotronik AG, Bülach, Switzerland

^b^Medtronic, Mansfield, MA, USA

^c^Cook Medical Inc, Bloomington, IN, USA

The methodology behind the 3D reconstruction of the arterial centerlines from 2D radiographic images has been extensively detailed elsewhere and will only be explained here briefly (Schumann et al. 2017). Following treatment, a pair of 2D X-ray angiographic images was acquired with the treated leg in supine and flexed positions (knee/hip flexion of approximately 70°/20°). Each pair was separated by an angle of at least 45°, which is required for the accurate reconstruction of the 3D geometry. The images were saved as DICOM files and transferred to a workstation for processing. The set of X-ray images at each leg position were first calibrated using the fiducials on the calibration phantom. The calibrated images were, then, transferred to a custom-built 3D reconstruction software, where the main branch of the arteries was traced with b-splines. Together with the calibration information, the points corresponding to the segmented lumen were triangulated in 3D space. These points were subsequently used to compute the 3D arterial centerline. Using each centerline point as a center, ellipses were fitted to the points that made up the lumen boundary along the length of the artery. As a final step, the individual ellipses were merged to generate the 3D model of the arterial lumen. The accuracy of the reconstructions was evaluated by calculating the forward- and backward-projection errors (Schumann et al. 2017). Although the results showed that the ellipsoid represented a good approximation of the arterial lumen, this simplified shape cannot precisely characterize some of the complex arterial deformations, such as pinching, that occur when the leg is bent. As a result, arterial pinching was designated as a significant decrease in the lumen diameter around the kinked regions.

For each patient, the reconstructed 3D geometries of the arterial lumen in straight and flexed positions were saved as surface STL models and imported into ANSYS ICEM CFD (ANSYS Inc., Pittsburg, USA) for meshing purposes. The arteries were primarily meshed with tetrahedral elements. In order to increase the accuracy of the results at the boundary, the mesh was inflated to incorporate prism elements toward the wall (Rikhtegar et al. 2012, 2013). The same inflation options were used for all models, with the number of layers, growth rate, and maximum thickness set as 15, 1.3, and 0.15 mm, respectively. A mesh sensitivity analysis was performed by changing the settings of the global tetrahedral mesh, while keeping the parameters of inflation constant. The influence of the mesh size on the velocity, pressure, and WSS parameters was evaluated at the locations of minimum and maximum lumen diameter, as well as at the segments with high curvature changes (Rikhtegar et al. 2013; Chiastra et al. 2015). Additionally, the number of iterations required for a timestep to converge was considered to be an important indicator that the mesh was satisfactory. Consequently, the mesh was refined until the maximal differences in the flow parameters between subsequent refinements were less than 4%, and the timesteps converged between 4 and 6 iterations (Rikhtegar et al. 2013; Chiastra et al. 2015; Liu et al. 2015). Depending on the lengths of the models, the ideal mesh size was found to be between 1.8 and 2.2 million elements, with approximately a 2:1 ratio between the number of prism and tetrahedral elements.

**Fig. 1:**
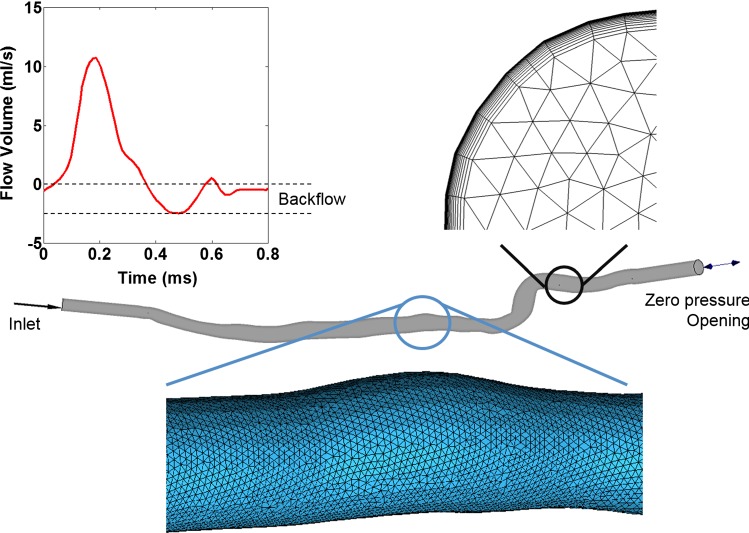
An example of the geometry, mesh, and boundary conditions used in the CFD analyses. The patient-specific geometry of an artery in the flexed leg position is presented in the middle (beige). The longitudinal view shows the tetrahedral surface mesh along the length of the artery (blue), while the cross-sectional view illustrates the mesh inflation toward the wall (black). An MRI-measured volumetric flow rate and a zero-pressure opening are applied as boundary conditions at the inlet and outlet, respectively (Mohajer et al. 2006)

The transient flow condition within the artery was simulated with ANSYS CFX 18.0 (ANSYS Inc., Pittsburg, USA). The blood was modeled as a Newtonian fluid (*ρ* = 1050 kg/m^3^; *µ* = 3.5 × 10^−3^ Pa s), and the flow was considered to be laminar. A volumetric flow profile obtained from an MRA measurement was applied at the inlet of the artery (Fig. 1; Mohajer et al. 2006). It had the characteristics of a triphasic flow and represented the flow behavior at the popliteal artery of an old patient with mild PAD. To account for the anatomical differences between patients, the magnitude of the velocity was calculated for each patient based on the inlet diameter of each arterial model. Additionally, the inlet was extended with a uniform cylinder until a fully developed flow profile was achieved at the initial position of the patient-specific segment. A zero-pressure condition was applied at the outlet of the artery (LaDisa et al. 2005; Wood et al. 2006; Rikhtegar et al. 2012, 2013; Desyatova et al. 2017; Migliori et al. 2017). In order to accommodate the backflow that was present in the inlet velocity, the outlet was set as an opening and was extended to avoid any boundary effects. The length of this extension was determined by monitoring the changes in the magnitude and direction of the flow velocity at the end of the patient-specific region. The artery wall was prescribed to have a no-slip condition. Three cardiac cycles were simulated and, to avoid transient effects, only the last cycle was evaluated. The target convergence criteria for each analysis were selected as 1 × 10^−5^, which represents fine convergence.

The hemodynamic behaviors of the arteries were investigated by evaluating the areas affected by time-averaged WSS (TAWSS) and OSI in the arterial wall. The adverse flow conditions that may trigger restenosis were adopted from the literature as low TAWSS (< 0.5 Pa; Nordgaard et al. 2010; Malek and Alper 2013; Dolan 2014), high TAWSS (> 7 Pa; Fukumoto et al. 2008), and high OSI (> 0.3; Desyatova et al. 2017). Due to significant differences between the geometries of each model, the results were not normalized with respect to the total luminal surface area, but were reported as the total area (cm^2^). Additional results in the form of mean TAWSS and OSI, as well as the 1st, 5th, 95th, and 99th percentile of these parameters, are given in Online Resource 1.

Paired and unpaired *t* tests were utilized to analyze the changes in the target areas due to leg flexion, treatment method, presence of kinking, and occurrence of restenosis. A *p* value below 0.05 was considered to be statistically significant. Furthermore, logistic regression analyses were used to predict the risk of restenosis from adverse hemodynamical effects. In total, three models were evaluated. The first two included either only non-flow-related patient characteristics or CFD-calculated flow parameters, and the final model incorporated parameters from both categories to create a mixed model. Following the prediction, a paired *t* test between the predicted values and clinical data was performed to assess whether the models produced statistically significant differences (*p* < 0.05) between restenosed and non-restenosed patients. The accuracy of each model was estimated from leave-one-out analyses and reported in percentage (%). The McFadden’s *R*^2^ was calculated to compare the predictive strength between the models. Finally, le Cessie–van Houwelingen–Copas–Hosmer unweighted sum of squares test was used to determine the goodness of fit of the models, with a p value below 0.05 being indicative of a poor fit. All the statistical analyses were conducted in the open-source package R 3.5.0.

## Results

Despite substantial changes in the arterial geometries during leg movement, none of the hemodynamical markers—low TAWSS, high TAWSS, and OSI—was significantly affected by the flexion of the leg (*p* values > 0.1). Nevertheless, for the majority of the patients, leg flexion resulted in an increase in the area that was affected by low TAWSS, a decrease in the area affected by high TAWSS, and a decrease in the area affected by high OSI (Table 2).

**Table 2:** Areas of the FP artery affected by low TAWSS (< 0.5 Pa), high TAWSS (> 7 Pa), and high OSI (> 0.3) in straight and flexed leg positions

Datasets	*N*	Areas affected by adverse flow behaviors (cm^2^; Mean ± Std)								
		Low TAWSS			High TAWSS			High OSI		
		Straight	Flexed	p*	Straight	Flexed	p*	Straight	Flexed	p*
Complete	20	1.8 ± 2.4	3.4 ± 3.6	0.113	0.6 ± 1.1	0.5 ± 0.9	0.587	6.5 ± 3.3	5.7 ± 3.5	0.486
Stent	10	0.9 ± 1.5	4.4 ± 4.6	**0.044**	0.5 ± 1.1	0.4 ± 1.0	0.795	9.0 ± 2.9	8.1 ± 3.5	0.532
PTA	10	2.7 ± 3.0	2.4 ± 2.0	0.789	0.8 ± 1.2	0.5 ± 0.8	0.628	4.0 ± 1.2	3.4 ± 1.1	0.256
p**		0.119	0.221		0.623	0.714		**< 0.001**	**0.002**	
Kinked	7	1.3 ± 1.7	5.9 ± 4.5	**0.045**	0.7 ± 1.3	0.5 ± 1.2	0.826	8.6 ± 2.6	8.7 ± 3.7	0.953
Non-kinked	13	2.1 ± 2.8	1.6 ± 2.0	0.954	0.6 ± 1.1	0.4 ± 0.7	0.608	5.4 ± 3.2	4.2 ± 2.1	0.269
p**		0.426	**0.048**		0.952	0.876		**0.029**	**0.016**	
Restenosis	7	2.6 ± 3.3	5.6 ± 4.3	0.180	0.7 ± 1.5	0.5 ± 1.0	0.685	7.5 ± 3.5	6.6 ± 3.6	0.631
No restenosis	13	1.4 ± 1.9	2.2 ± 2.6	0.351	0.6 ± 1.0	0.5 ± 0.8	0.743	5.9 ± 3.3	5.3 ± 3.4	0.618
p**		0.372	0.097		0.807	0.982		0.340	0.440	

Bold indicates statistically significant values

The area is reported for the complete dataset, as well as for groups defined by different treatment methods, presence of kinking observed in the flexed leg positions, and clinical outcome. *N* denotes the number of patients for each group, while p* and p** represent the outcomes of the *t* tests between different leg positions and different groups, respectively

Separately analyzing the patients within groups defined by the treatment method revealed significant effects associated with adverse flow behaviors. For stented arteries, leg flexion caused a statistically significant difference in the areas affected by low TAWSS (*p* = 0.044), whereas no difference was observed for the patients that only underwent PTA (*p* = 0.789; Table 2). Additionally, the treatment method was found to have a significant effect on the change in the areas affected by low TAWSS due to leg flexion (stent: 3.47 cm^2^ ± 4.46 cm^2^ vs. PTA: − 0.31 cm^2^ ± 1.38 cm^2^, *p* = 0.028). Furthermore, the area affected by high OSI was significantly higher for stented arteries than dilated ones for both straight (*p* < 0.001) and flexed (*p* = 0.002) positions.

Similarly, grouping the datasets with respect to the presence of kinking observed in the flexed leg positions showed that the kinked arteries had a statistically significant increase in the area affected by low TAWSS between different leg positions (*p* = 0.045). On the other hand, leg flexion had no effect on this specific hemodynamical marker for non-kinked arteries (*p* = 0.954). In the flexed position, the area affected by low TAWSS was significantly higher in arteries that exhibited kinking (*p* = 0.048), but no differences in any of the markers were observed in the straight configuration. In addition, the presence of arterial kinks significantly increased the average area affected by high OSI in the flexed position (*p* = 0.016).

For patients grouped according to the presence or absence of restenosis at 6 months, leg flexion did not cause any statistically significant differences in the flow parameters (Table 2). Moreover, no significant differences were found in any of the hemodynamical markers between the two groups for either leg position. Despite these observations, the areas affected by low TAWSS increased with leg flexion and were higher for restenosed arteries. Similarly, regardless of the leg position, the areas under high OSI were higher for patients diagnosed with restenosis. In contrast, the areas associated with high TAWSS were affected by neither the flexion of the leg nor the occurrence of restenosis.

**Fig. 2:**
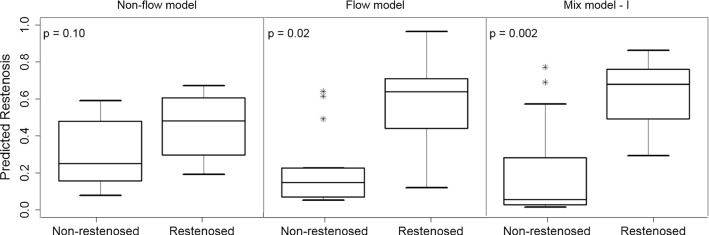
The prediction of restenosis based on the logistic regression of the non-flow (left), flow (middle), and mixed model-I right). The latter two showed a statistically significant difference between the restenosed and non-restenosed patients. The mean difference between the predicted values for the two groups was larger for the mixed model, resulting in a more significant *p* value when treatment method was considered as an additional predictor in the model

Three logistic regression models were built to investigate the relationship between the occurrence of restenosis and adverse hemodynamics in the FP arteries. In the first model, all hemodynamical markers were excluded to evaluate the potential of patient and treatment characteristics to predict restenosis (i.e., the non-flow model; Table 3). This model included the treatment method, lesion length, the presence of kinking, age, and the level of calcification, but it was unable to produce a statistically significant difference between restenosed and non-restenosed arteries either individually or collectively (*p* = 0.10; Fig. 2).

**Table 3:** The main results from the logistic regression analyses of the non-flow, flow, mixed model-I (flow + treatment method), and mixed model-II (flow + kinking + lesion length + age + plaque morphology)

Models	*p*	Acc. (%)	AUC	*R* ^2^
Non-flow model	0.10	0.65	0.75	0.13
Flow model	0.02	0.80	0.85	0.29
Mixed model-I	0.002	0.80	0.87	0.38
Mixed model-II	0.01	0.75	0.87	0.33

The table reports, for each model, the statistical significance between the restenosed and non-restenosed patients based on the prediction of the model (*p* value); the leave-one-out accuracy (Acc. in %); the area under the curve (AUC); and the McFadden’s pseudo-*R*^2^. The non-flow parameters were not able to predict the restenosis at 6 months (non-flow model), while a model based solely on flow markers provided a statistical difference between the two groups (flow model). Moreover, the inclusion of the treatment method to the flow model as an additional predictor (mixed model-I) was able to improve the prediction, which was not the case with the other non-flow predictors (mixed model-II)

In the second model, only the hemodynamical parameters were used to predict the risk of restenosis (i.e., the flow model). Before building the model, it was found that the high TAWSS parameters for both leg positions were significantly multicollinear with variance of inflation of 22 and 18 for straight and flexed configurations, respectively. Similarly, the high OSI parameters showed multicollinearity between the two leg positions. Consequently, the high TAWSS and high OSI in the flexed position were excluded from the model, which, therefore, only included the low TAWSS in straight and flexed positions, as well as the high TAWSS and high OSI in the straight configuration as predictors. This model was able to predict the risk of restenosis with an accuracy of 80% and showed a statistically significant difference between restenosed and non-restenosed arteries (*p* = 0.02; Table 3).

As a final step, the non-flow parameters were added to the flow model one at a time. With the inclusion of the treatment method (i.e., the mixed model-I), the possibility of restenosis became strongly statistically significant between the two groups (*p* = 0.002; Fig. 2), and the addition produced a more optimal fit than the flow model (Table 3). The p value corresponding to the goodness of fit was 0.23, suggesting no evidence of poor fit. The inclusion of the remaining predictors to this modified model violated the multicollinearity assumptions. As such, the parameters of kinking, lesion length, age, and plaque morphology were added to the flow model separately from the treatment method (i.e., the mixed model-II). However, their inclusion did not improve the statistics (*p* value = 0.01, Acc. = 75%).

## Discussion

The primary method of endovascular treatment in the FP artery has constantly evolved over the last two decades (Norgren et al. 2007). However, despite the rise of new technologies, such as drug-coated devices, the long-term outcomes of endovascular treatment in peripheral arteries seem to have reached a plateau, with controlled clinical studies consistently showing restenosis rates between 15 and 20% and an approximate TLR rate of > 10% (Micari et al. 2018). The high rate of restenosis is believed to originate from repetitive mechanical deformations of the treated arteries during leg flexion (Klein et al. 2009; MacTaggart et al. 2014; Gökgöl et al. 2017). However, the effects of the post-treatment arterial deformations on the flow behaviors of the FP arteries remain unknown. Therefore, this study aimed to build a personalized characterization of the hemodynamic environment of the FP arterial segment and to evaluate the ability of hemodynamic markers to predict restenosis observed at 6-month follow-up.

**Fig. 3:**
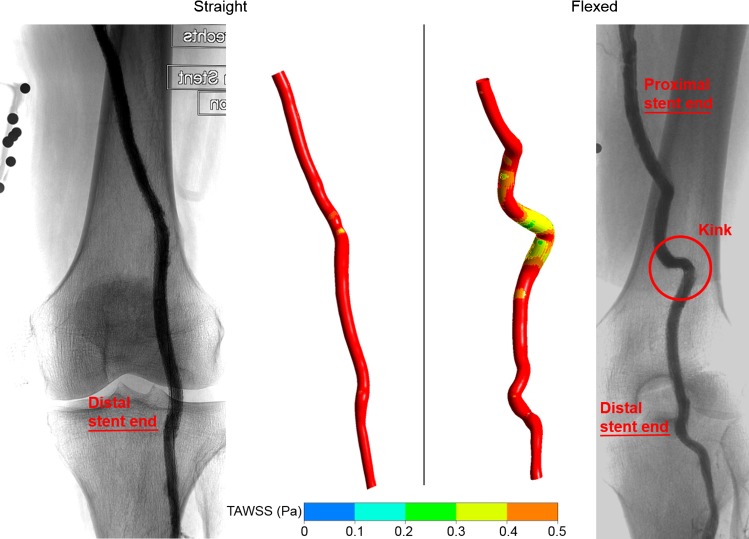
The TAWSS distribution in a stented artery that exhibited arterial kinking during leg flexion. The X-ray images were acquired in the straight and flexed leg positions and show the locations of the stented region and the arterial kink when the leg is flexed. The location of the atheroprone areas described by the TAWSS < 0.5 Pa were concentrated around the vicinity of the kink or highly curved segment

The analysis of the adverse flow conditions on the entire dataset showed that the flexion of the leg has no significant effects on any of the hemodynamical markers (Table 2). However, notable differences between the two treatment modalities were observed. While leg flexion did not change the hemodynamics following PTA, the same movement was found to significantly modify certain flow behaviors in stented arteries. This observation can be connected to previous findings, which showed that stent implantation limits the axial shortening of the artery during leg flexion and, therefore, induces arterial kinking when the leg is flexed (Arena 2005; Gökgöl et al. 2017). Kinking is not only associated with an important increase in the local arterial curvature, but also leads to contractions in the diameter of the artery (i.e., pinching), especially in the presence of stent struts. In fact, these radial deformations may constitute a more objective characterization of the adverse conditions as they can be accurately reconstructed from 2D images, whereas kinking represents a subjective assessment of the arterial deformation that does not follow any established criteria. Nevertheless, the numerical approach used in this study was able to simultaneously capture the effects associated with the local changes in curvature and pinching and showed that they are responsible for an important increase in the atheroprone areas along the artery wall (Fig. 3). As such, the analyses confirmed that the stented and/or kinked arteries are more susceptible to experiencing adverse hemodynamics due to a decreased flow velocity during leg flexion. Additional results on the entire dataset, which further support this statement, are given in Online Resource 2.

Individually, none of the hemodynamical markers were able to explain the occurrences of restenosis at 6-month follow-up. While the area affected by low TAWSS was the parameter showing the largest difference between patients with and without restenosis, even this difference was not significant. However, the combination of different hemodynamical predictors (flow model) was able to determine the risk of restenosis with an accuracy of 80%, which was notably higher compared to the prediction based solely on clinical parameters (i.e., non-flow model; Table 3).

The ability of the flow model to predict the 6-month outcome (TLR) is improved when the treatment method is included as an additional predictor (Fig. 2). This indicates that the effects of the treatment on the flow parameters were not fully captured by the CFD simulations. A possible explanation for this may be the insufficient resolution of the imaging system. The radiologic images used to reconstruct the arterial tract were able to capture the macroscopic shape of the artery and the major changes in the arterial lumen, but not the submillimetric stent geometry. As such, the CFD simulations are unable to accurately determine the local modifications on the flow condition caused by the presence of the stent. In that sense, an improved image acquisition based on intra-arterial imaging methods could provide a more detailed picture of the stent/wall apposition and therefore provide the basis for more accurate CFD simulations.

The comparison of the current results with previous studies is challenging due to the limited number of numerical works on patient-specific FP arteries and the absence of investigations on the hemodynamic implications of arterial deformations under leg flexion. Additionally, the differences in subject characteristics, age groups, boundary conditions, and the representation of the flow parameters make it even more difficult to draw quantitative comparisons between them. Nevertheless, some qualitative similarities can be observed. Wood et al. (2006) performed CFD simulations based on the MRI images of 18 young and healthy FP arteries in a straight position. Similar to our findings, their analyses showed that low TAWSS and high OSI values were concentrated within and distal to the native curvature in the FP arteries. Xu et al. (2016) built CFD models based on the MRI images of 14 vessels obtained from seven patients in supine position. Although they did not specifically report any parameters related to adverse hemodynamics, they concluded that the native curvature of the FP arteries can lead to important variations in the WSS parameters along the length of the artery, which corresponds well with our observations. Finally, Desyatova et al. (2017) conducted CFD analyses based on patient-specific arterial centerlines obtained from seven different age groups. Unlike the aforementioned studies, the centerlines corresponded to a gardening position. The 3D surface models of the lumen were generated using an idealized cross section that did not include any patient-specific information. Their results suggested that the arteries of old subjects were afflicted with low TAWSS not only at the local curvature zones, but along the entire length of the artery. This high presence of adverse hemodynamics can be attributed to the higher flexion angle, but it can also be a product of the study’s limitations related to the generation of 3D models.

There are also a handful of clinical investigations, which computed the WSS parameters of healthy and stented arteries based on duplex US measurements. Schlager et al. (2011) obtained velocity measurements in the FP arteries of 46 healthy subjects in supine and sitting positions. Their calculations showed that the mean WSS significantly decreased when the patients were sitting. This agrees well with our findings, which show an increased presence of low TAWSS when the leg is flexed. In a following study, they applied the same methodology to measure the peak and mean WSS values of 87 patients with PAD following nitinol stent implantation (Schlager et al. 2014). Within the stented segment, they reported a mean WSS of 1.5 Pa and a peak WSS of 3.1 Pa in the supine leg position. This correlates well with our mean and peak values of 1.2 Pa ± 0.4 Pa and 3.5 Pa ± 0.9 Pa, respectively. The differences could be due to patient and stent characteristics, as well as due to the inherent limitations associated with both approaches.

The current study has several limitations. First, the personalization of the numerical flow simulations results from the 3D reconstruction of the arterial lumen profile from orthogonal radiographic images. This approach has the benefit of being easily integrated into the clinical workflow, but it is also limited by the precision of the imaging system. As such, localized arterial bending/pinching might not always be visible on the radiographic projections. Furthermore, actual pinching of the artery during leg flexion cannot be accurately represented because of the simplified shape of the elliptical cross section. 2D X-ray angiography is not suitable for reconstructing the arterial wall, the side branches, and any of the complications that might occur following PTA or stent implantation, such as arterial dissections, residual stenosis, stent malappositions, and migrations. In vivo imaging methods, like optical coherence tomography (OCT), need to be utilized in order to incorporate these features into the 3D models, which would increase the accuracy of the CFD analyses and predictions. Due to limited information regarding wall thickness and plaque morphology, as well as limited availability of the stent designs, CFD analyses were conducted without stent models. Consequently, the analyses ignored the influence of the stents on the flow, which may impact the accuracy of the simulations. Moreover, the lack of stent models in the numerical framework prevents the analysis of arterial damage that originates from mechanical interactions between stents and arteries during leg flexion. Since the formation of restenosis depends on multiple factors, which include structural and hemodynamical parameters, this is an open point that needs to be addressed in future studies. Despite this limitation, results showed expected differences between the two treatment methods, suggesting that our analyses were able to partially capture the disruptions in the flow caused by the presence of stent struts.

Another limitation concerns the lack of patient-specific boundary conditions at the inlet and outlet of the models. The velocity profile at the inlet corresponds to a reference measurement on this arterial segment and enables the evaluation of the hemodynamic markers in an equivalent situation for all patients. Alternative approaches could rely on ultrasonic flow measurements of each patient, but these measurements would only describe the situation at a single time point that might not reflect the average daily flow patterns of the patients. Moreover, care was taken to sufficiently extend the models at both the inlet and outlet to limit unrealistic boundary effects. The CFD analyses were performed within a rigid framework, meaning that the displacement of the arterial wall by the blood was overlooked. However, this approach is commonly used in peripheral arteries (Wood et al. 2006; Xu et al. 2016; Desyatova et al. 2017), and previous studies have shown that the inclusion of structural parameters such as arterial compliance only slightly changes the flow behaviors in the femoral bifurcations (Kim et al. 2008). To our knowledge, no existing studies suggest that fluid–structure interactions must be considered for accurate simulations that involve FP arteries. Finally, the occurrences of restenosis observed at 6-month follow-up could be the result of damage exerted onto the artery during endovascular therapy. However, there is no discernible way to definitively identify the mechanisms behind the causes of restenosis using conventional methods of X-ray angiography and duplex ultrasound. In vivo imaging methods performed immediately after the intervention can be used to have strong indications for the causes of restenosis in each patient, but even these methods would not be able to exactly define whether restenosis is a product of in-stent restenosis (ISR), atherosclerosis, or a combination of both. As such, it would be equally incorrect to rule out atherosclerosis as the process behind restenosis. However, further support for this statement is critical to ensure the validity of our initial hypothesis and conclusions. To that end, additional follow-ups should be scheduled to cover a longer period. Arterial imaging should also be collected on patients that exhibit restenosis to derive direct correlations between CFD-calculated results and clinical observations.

This study showed that arterial kinks occurring due to leg flexion invite poor hemodynamic behaviors, but that kinking is not sufficient to predict restenosis. Restenosis results from a combination of complex deformations, which includes not only kinking, but also the local variations in the lumen diameter and curvature. Incorporating this information into patient-specific models showed that atheroprone areas calculated using CFD simulations can provide insights into the risk of restenosis more successfully than traditional clinical markers. Since the hemodynamic parameters were calculated based on the immediate postoperative configuration of the artery, this approach has the potential to identify patients at increased risk of restenosis immediately after treatment. Based on this information, clinicians could take preventive measures, such as post-dilation, to improve the results and perform intensified secondary prevention to avoid additional complications.

## Electronic supplementary material

Below is the link to the electronic supplementary material.

Supplementary material 1 (PDF 101 kb)

Supplementary material 2 (PDF 6652 kb)
