# Supplementary material for: Prediction of restenosis based on hemodynamical markers in revascularized femoro-popliteal arteries during leg flexion
Source: Biomech Model Mechanobiol. 2019 Jun 13;18(6):1883–93. doi: 10.1007/s10237-019-01183-9 (PMC6825029; doi:10.1007/s10237-019-01183-9)
Supplement: Supplementary file 1 — Supplementary material 1 (PDF 101 kb) [file 10237_2019_1183_MOESM1_ESM.pdf]

Prediction of restenosis based on hemodynamical markers in revascularized femoro-popliteal arteries during leg flexion

Can Gökgöl, PhD; Nicolas Diehm, MD, MBA; Lorenz Räber, MD, PhD; and Philippe Büchler, PhD*

Biomechanics and Modeling in Mechanobiology

*Corresponding author**:** Assoc. Prof. Dr. Philippe Büchler

ARTORG Center for Biomedical Engineering Research, University of Bern, Bern, Switzerland

Email: philippe.buechler@artorg.unibe.ch; Phone: +41 31 631 5947

Table 1: The 1st, 5th, 95th, and 99th percentile, and the mean TAWSS (Pa) in straight and flexed leg configurations. The values are averaged over and reported for the complete dataset, as well as for groups defined by different treatment methods, presence of kinking observed in the flexed leg positions, and clinical outcome. N denotes the number of patients for each group.

**Datasets**	**N**	**TAWSS (Pa)**									
		**Straight**					**Flexed**				
		**Percentile**				**Mean**	**Percentile**				**Mean**
		**1st**	**5th**	**95th**	**99th**		**1st**	**5th**	**95th**	**99th**	
**Complete**	20	0.58 ± 0.15	0.75 ± 0.23	4.41 ± 2.64	6.29 ± 3.76	1.97 ± 0.91	0.57 ± 0.20	0.74 ± 0.29	3.90 ± 2.20	5.68 ± 3.10	1.86 ± 0.98
**Stent**	10	0.49 ± 0.07	0.61 ± 0.1	3.29 ± 2.61	4.46 ± 3.28	1.46 ± 0.78	0.32 ± 0.09	0.44 ± 0.16	2.93 ± 2.35	4.53 ± 3.42	1.36 ± 0.65
**PTA**	10	0.68 ± 0.15	0.92 ± 0.22	5.70 ± 2.15	7.73 ± 3.38	2.53 ± 0.72	0.71 ± 0.13	0.92 ± 0.17	4.97 ± 1.48	6.96 ± 2.22	2.42 ± 0.50
**Kinked**	7	0.46 ± 0.07	0.58 ± 0.10	3.91 ± 2.85	5.20 ± 3.74	1.74 ± 0.92	0.30 ± 0.09	0.41 ± 0.17	3.12 ± 2.85	4.50 ± 4.06	1.40 ± 1.29
**Non-kinked**	13	0.65 ± 0.14	0.86 ± 0.22	4.94 ± 2.26	7.10 ± 3.33	2.24 ± 0.82	0.67 ± 0.15	0.85 ± 0.20	4.35 ± 1.69	6.37 ± 2.30	2.14 ± 0.68
**Restenosis**	7	0.50 ± 0.08	0.61 ± 0.13	3.66 ± 2.94	5.04 ± 3.93	1.59 ± 0.94	0.42 ± 0.14	0.53 ± 0.19	3.02 ± 2.04	4.20 ± 2.82	1.36 ± 0.82
**No-Restenosis**	13	0.63 ± 0.16	0.84 ± 0.24	4.85 ± 2.47	7.02 ± 3.63	2.18 ± 0.86	0.65 ± 0.19	0.86 ± 0.29	4.41 ± 2.20	6.54 ± 3.03	2.16 ± 0.98

Table 2: The 1st, 5th, 95th, and 99th percentile, and the mean OSI in straight and flexed leg configurations. The values are averaged over and reported for the complete dataset, as well as for groups defined by different treatment methods, presence of kinking observed in the flexed leg positions, and clinical outcome. N denotes the number of patients for each group.

Datasets	N	OSI									
		Straight					Flexed				
		Percentile				Mean	Percentile				Mean
		1st	5th	95th	99th		1st	5th	95th	99th	
**Complete**	20	0.06 ± 0.01	0.09 ± 0.01	0.40 ± 0.02	0.46 ± 0.01	0.22 ± 0.02	0.05 ± 0.01	0.07 ± 0.01	0.39 ± 0.03	0.45 ± 0.02	0.20 ± 0.02
**Stent**	10	0.06 ± 0.01	0.09 ± 0.01	0.41 ± 0.02	0.46 ± 0.01	0.22 ± 0.01	0.04 ± 0.01	0.07 ± 0.01	0.40 ± 0.03	0.46 ± 0.02	0.21 ± 0.02
**PTA**	10	0.06 ± 0.01	0.09 ± 0.02	0.39 ± 0.02	0.45 ± 0.01	0.21 ± 0.01	0.05 ± 0.01	0.07 ± 0.02	0.38 ± 0.03	0.45 ± 0.02	0.19 ± 0.01
**Kinked**	7	0.05 ± 0.01	0.08 ± 0.01	0.41 ± 0.02	0.46 ± 0.01	0.22 ± 0.01	0.04 ± 0.01	0.07 ± 0.01	0.41 ± 0.03	0.46 ± 0.02	0.21 ± 0.03
**Non-kinked**	13	0.06 ± 0.01	0.09 ± 0.02	0.39 ± 0.02	0.46 ± 0.01	0.21 ± 0.02	0.05 ± 0.01	0.07 ± 0.02	0.38 ± 0.03	0.45 ± 0.01	0.20 ± 0.02
**Restenosis**	7	0.06 ± 0.01	0.09 ± 0.01	0.41 ± 0.02	0.46 ± 0.01	0.22 ± 0.01	0.05 ± 0.01	0.07 ± 0.01	0.40 ± 0.03	0.46 ± 0.02	0.20 ± 0.01
**No-Restenosis**	13	0.06 ± 0.01	0.09 ± 0.02	0.40 ± 0.02	0.45 ± 0.01	0.22 ± 0.02	0.04 ± 0.01	0.07 ± 0.02	0.39 ± 0.03	0.45 ± 0.02	0.20 ± 0.02
