# Supplementary material for: Prediction of restenosis based on hemodynamical markers in revascularized femoro-popliteal arteries during leg flexion
Source: Biomech Model Mechanobiol. 2019 Jun 13;18(6):1883–93. doi: 10.1007/s10237-019-01183-9 (PMC6825029; doi:10.1007/s10237-019-01183-9)

Prediction of restenosis based on hemodynamical markers in revascularized femoro-popliteal arteries during leg flexion

Can Gökgöl, PhD; Nicolas Diehm, MD, MBA; Lorenz Räber, MD, PhD; and Philippe Büchler, PhD*

Biomechanics and Modeling in Mechanobiology

*Corresponding author: Assoc. Prof. Dr. Philippe Büchler

ARTORG Center for Biomedical Engineering Research, University of Bern, Bern, Switzerland;
Email: philippe.buechler@artorg.unibe.ch

Figures 1-20: The TAWSS distribution in straight and flexed arteries for the entire dataset. The accompanying X-ray images show the locations of stented and dilated (PTA) regions, as well as arterial kinks when the leg is flexed. The location of the atheroprone areas described by TAWSS < 0.5 Pa were concentrated around the vicinity of the kinks or highly curved segments. Additional areas affected by adverse flow conditions were within the stented segments, in which leg flexion resulted in the intermittent pinching of the artery around the kinks and along the length of the stents.

Patient 1 – Stent / Restenosis

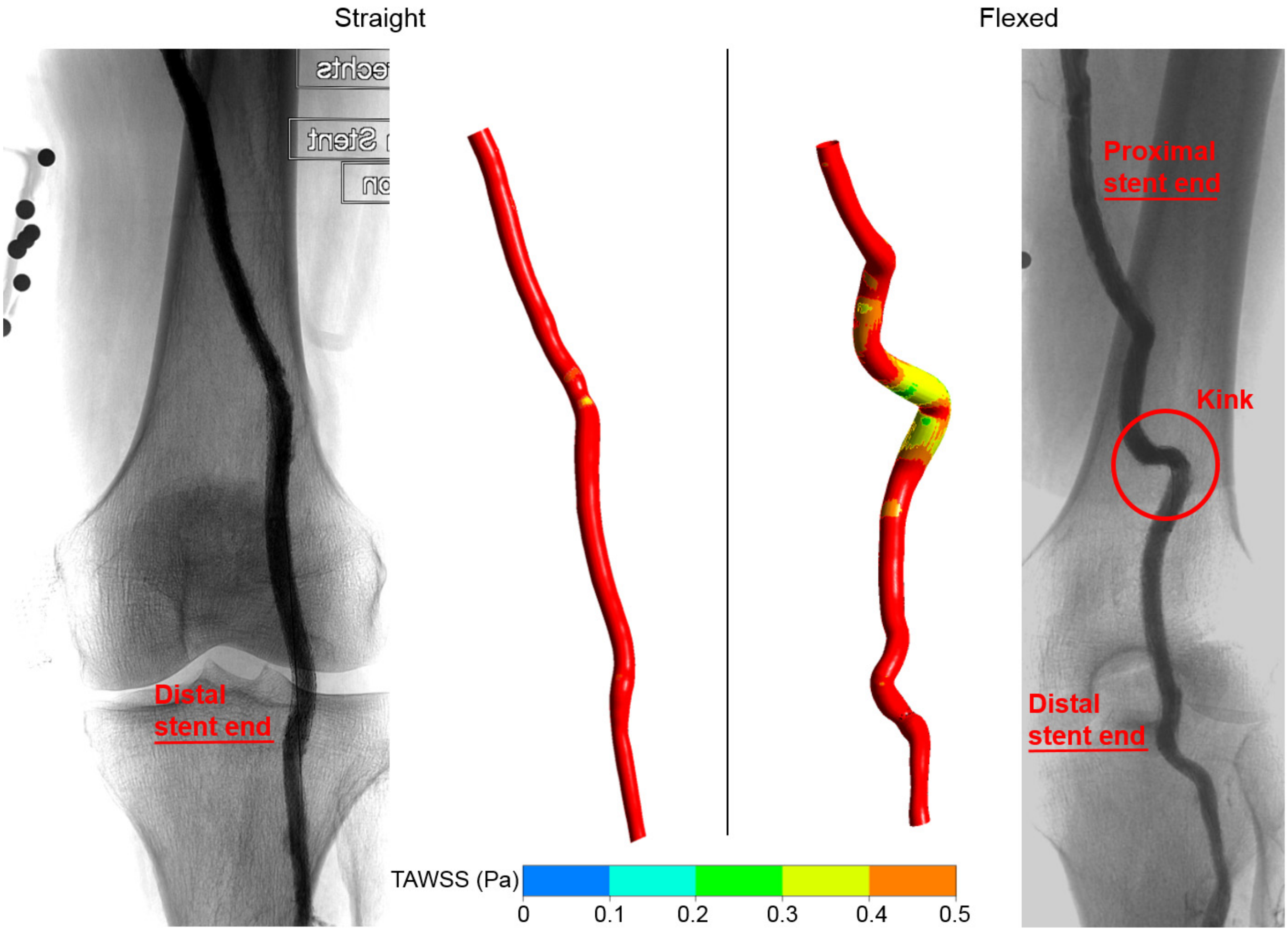

Patient 2 – Stent / Restenosis

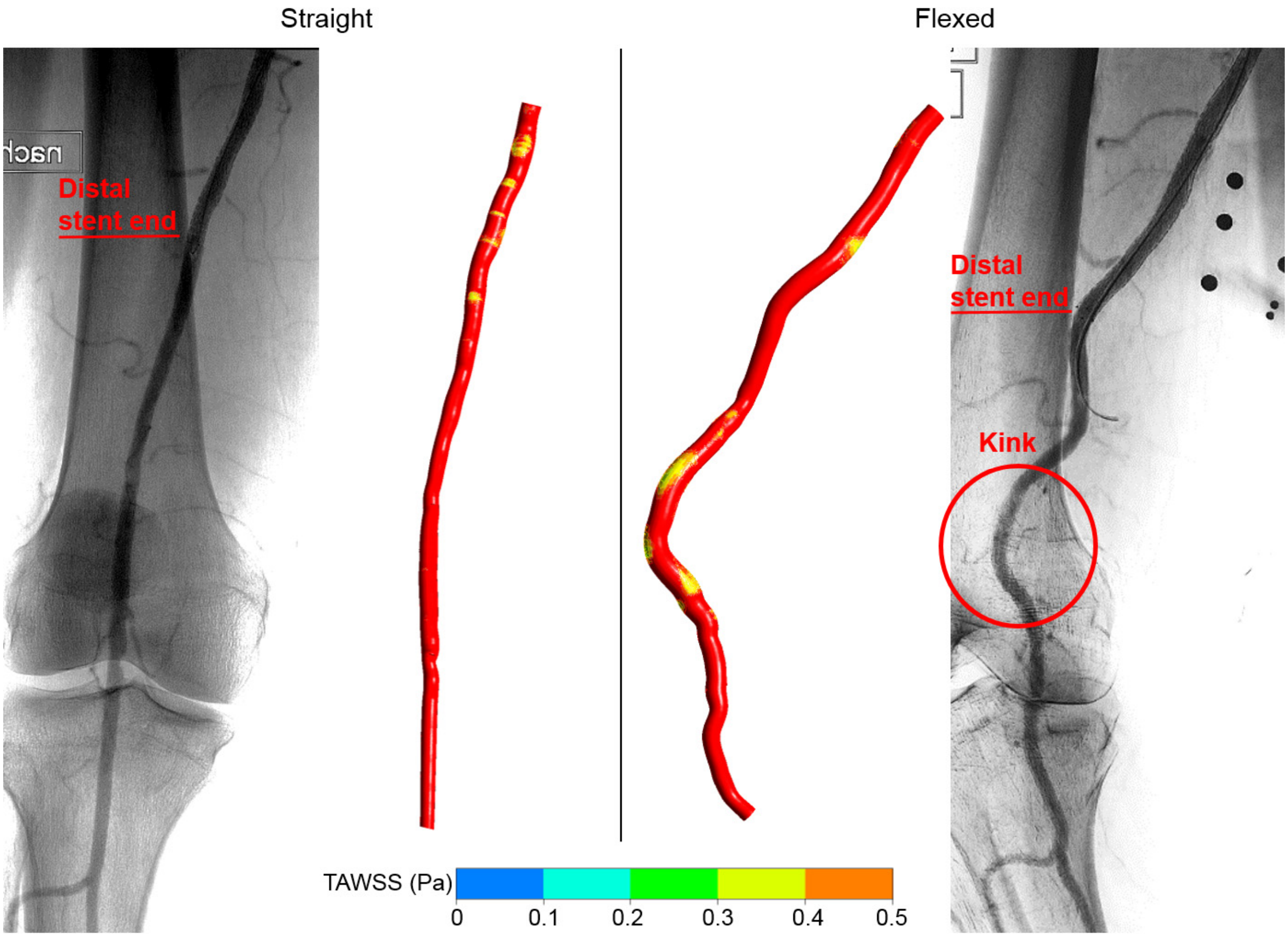

Patient 3 – Stent / Restenosis

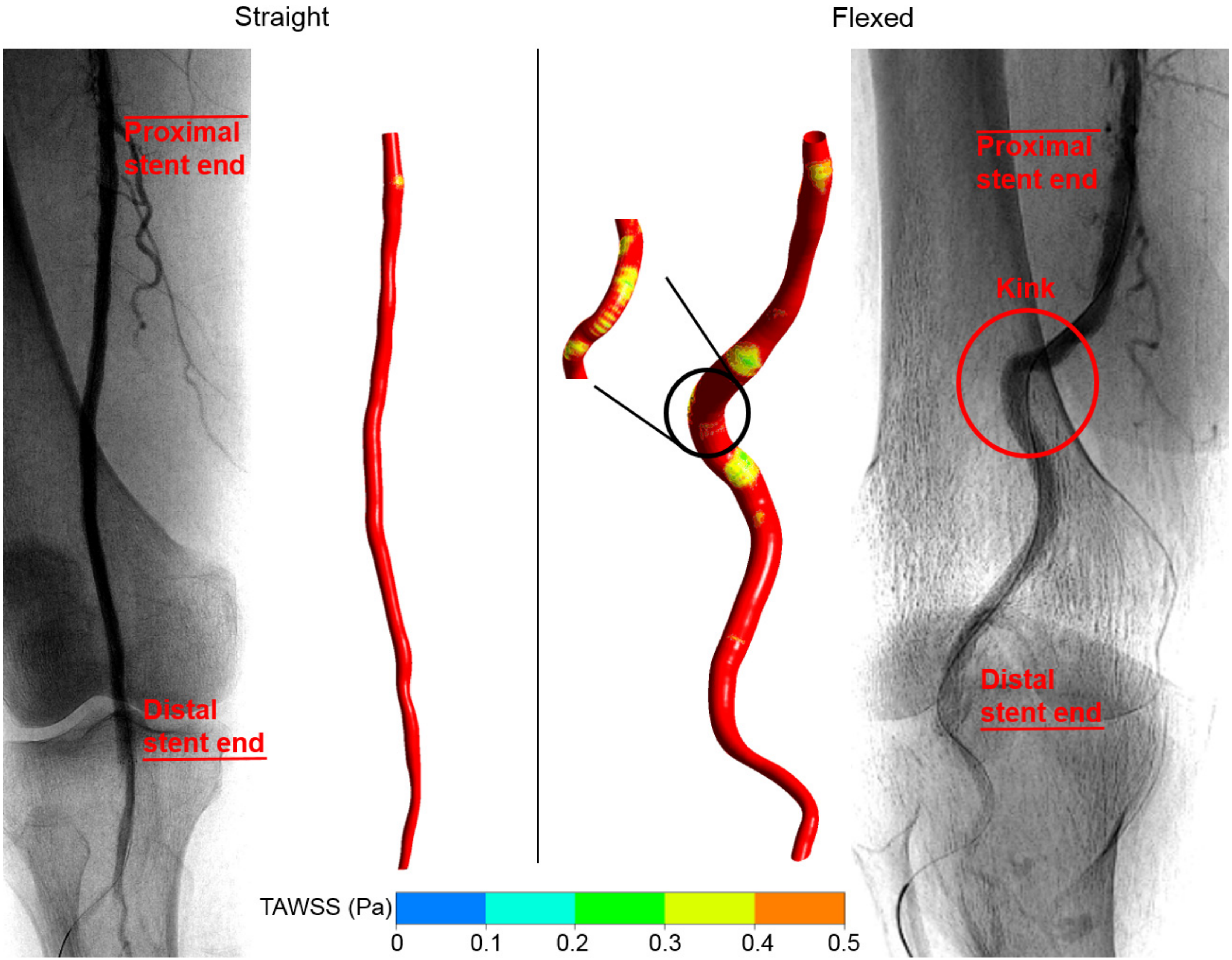

Patient 4 – Stent / Restenosis

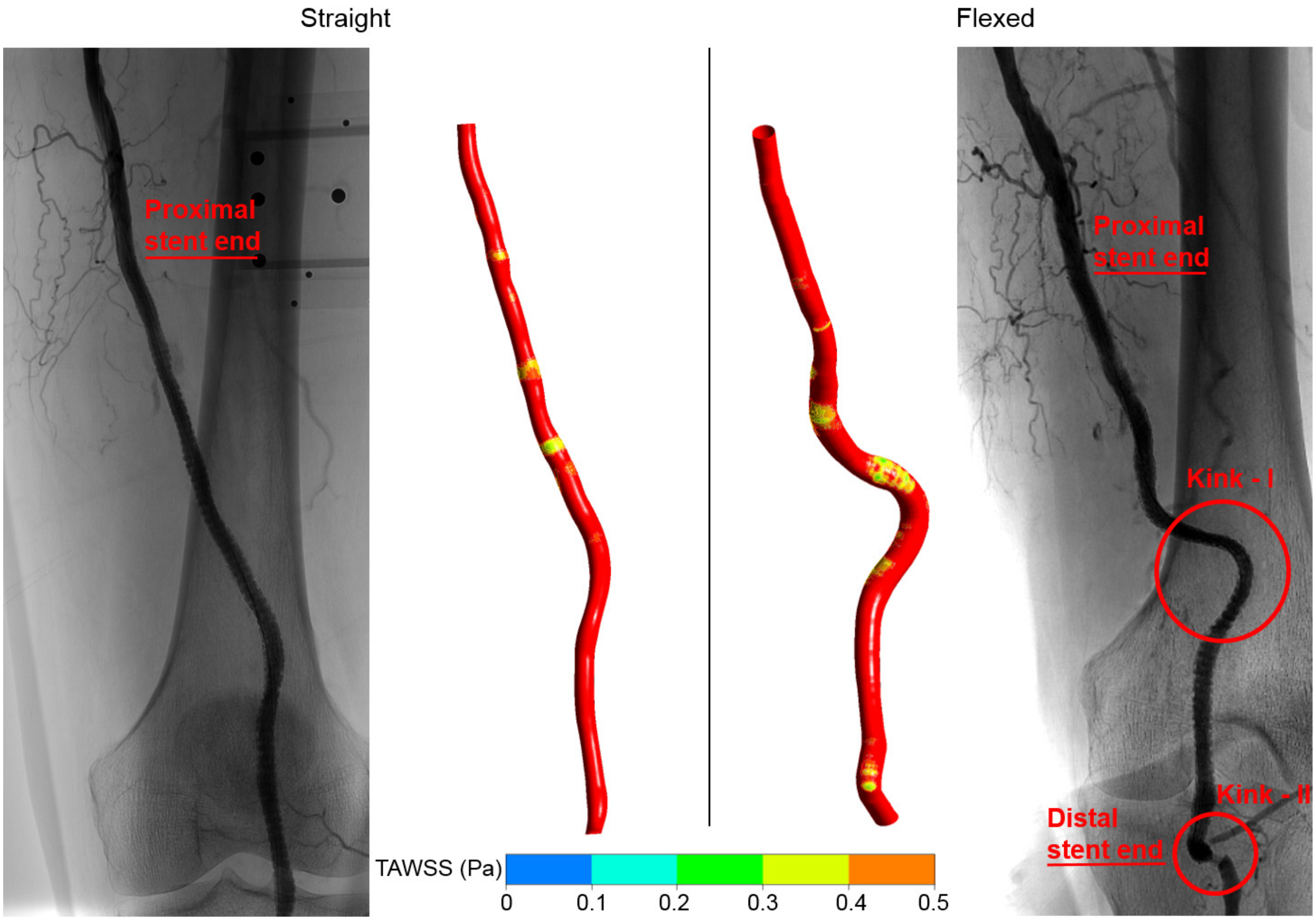

Patient 5 – Stent / Restenosis

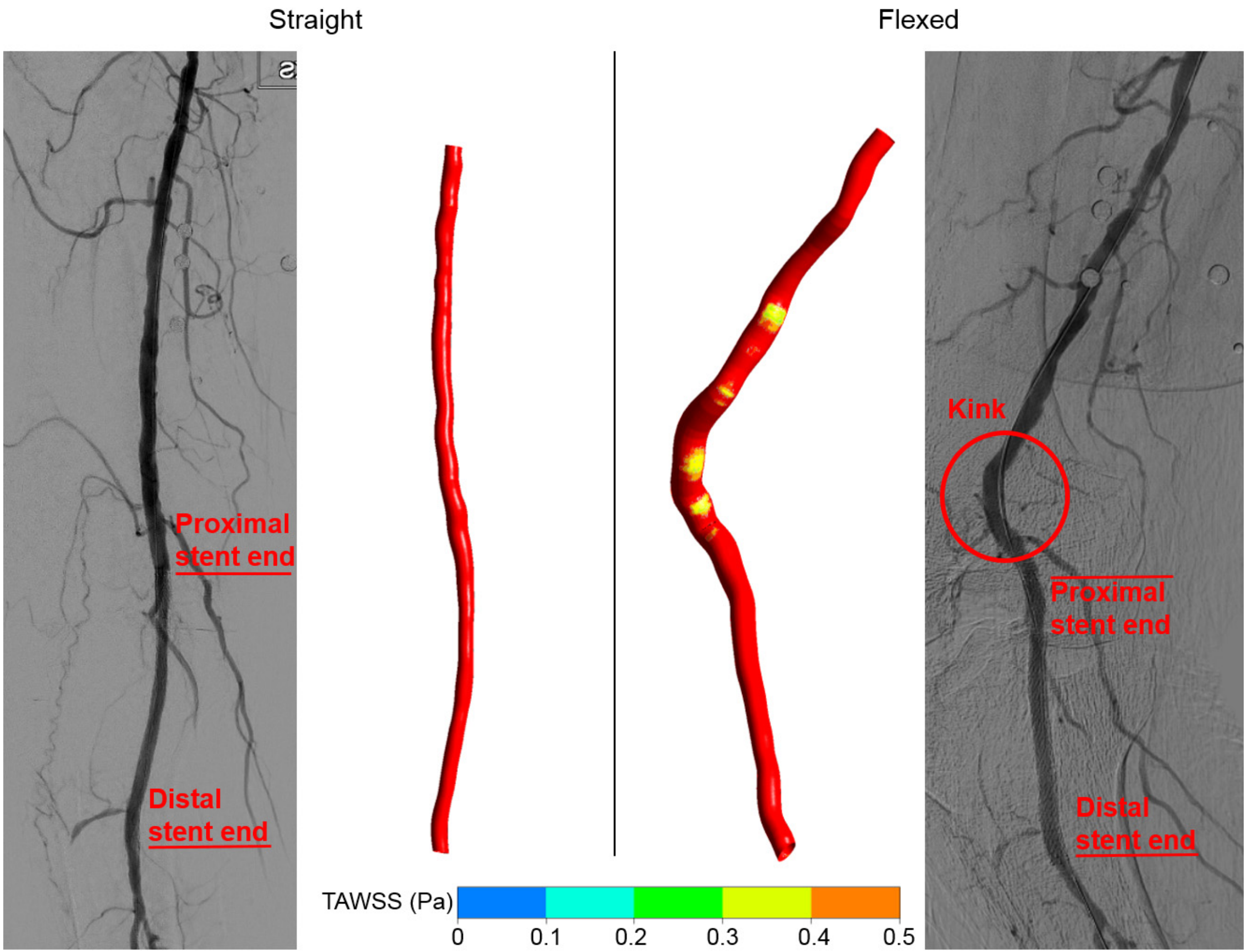

Patient 6 – Stent

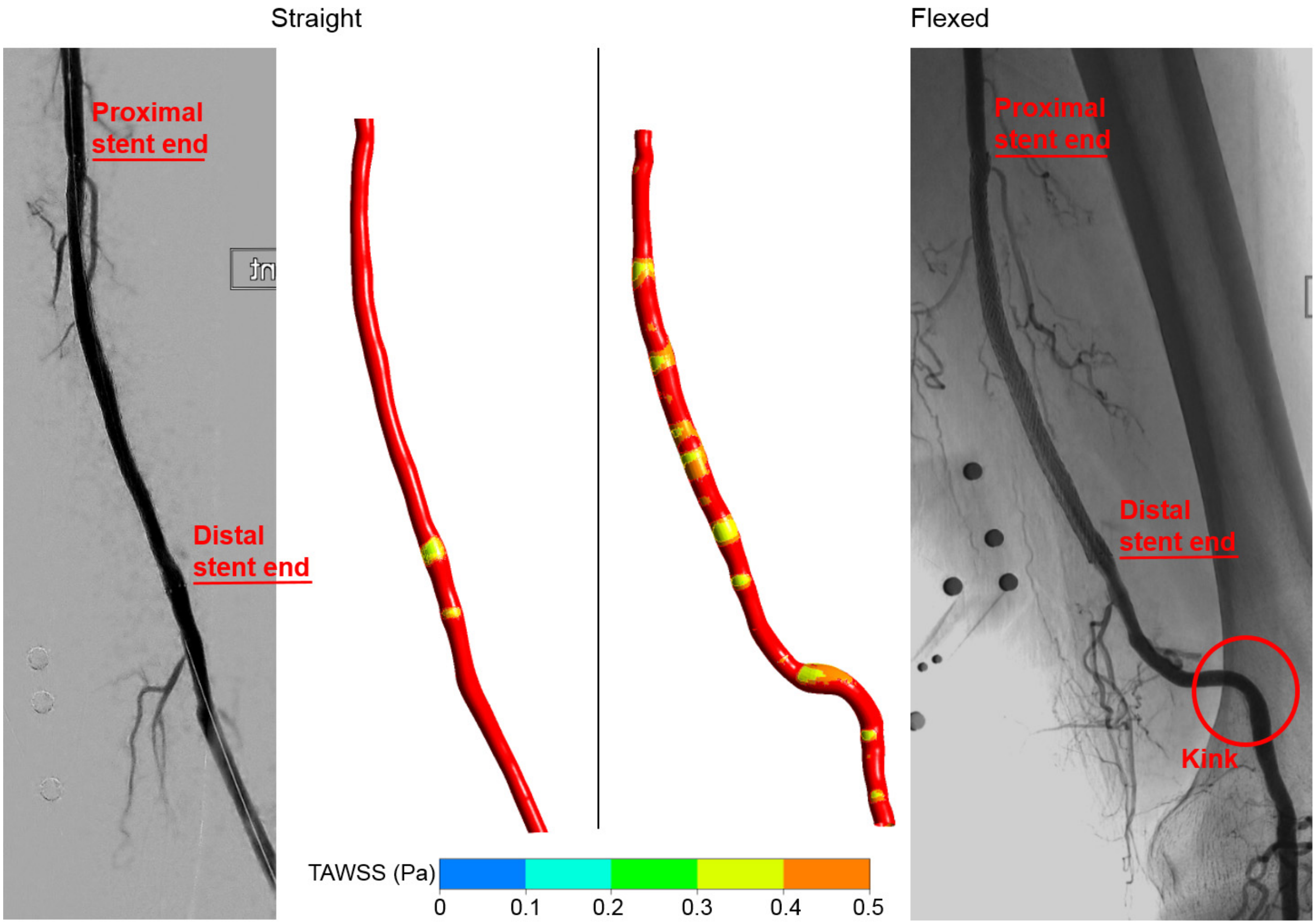

Patient 7 – Stent

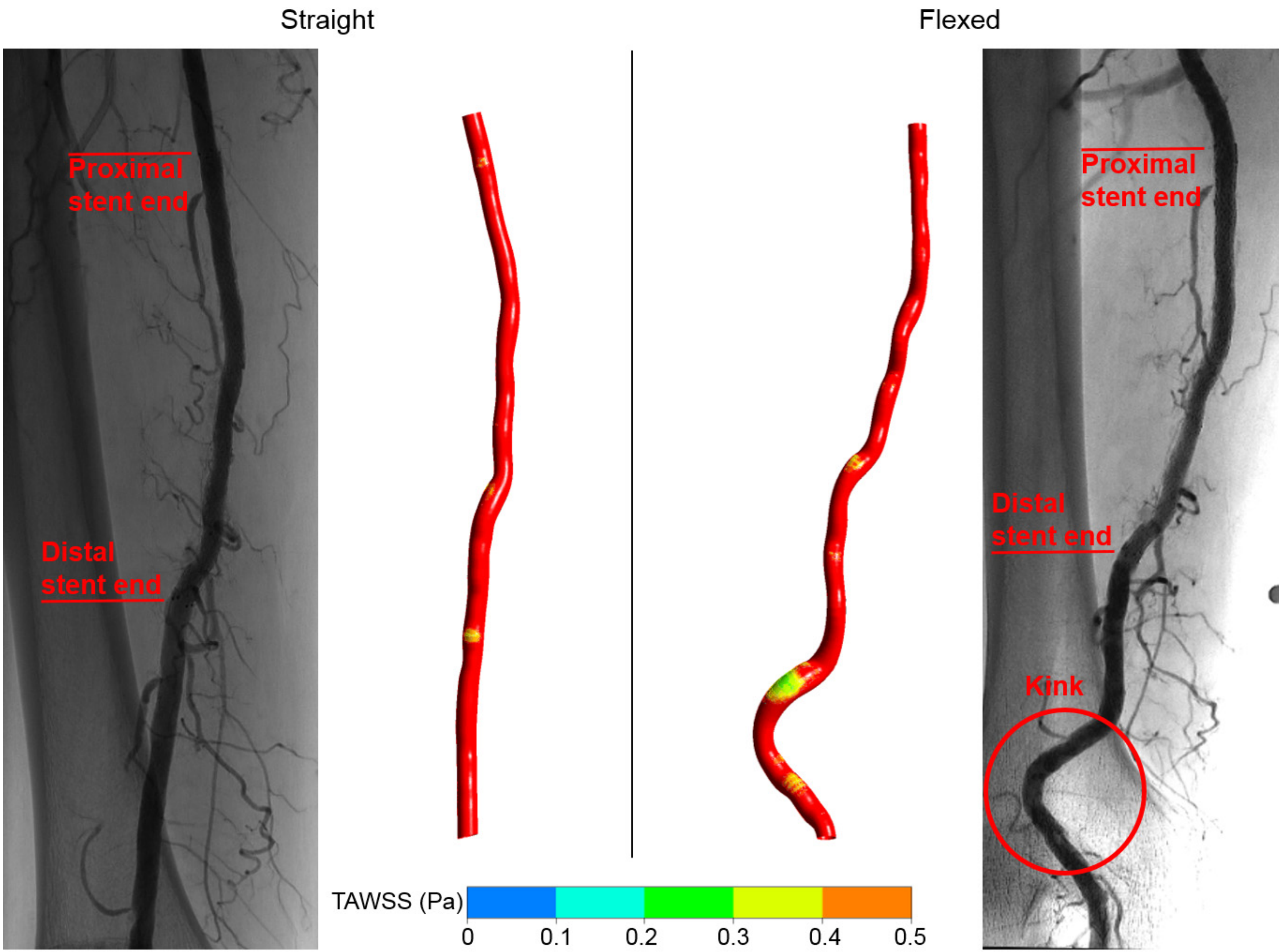

Patient 8 – Stent

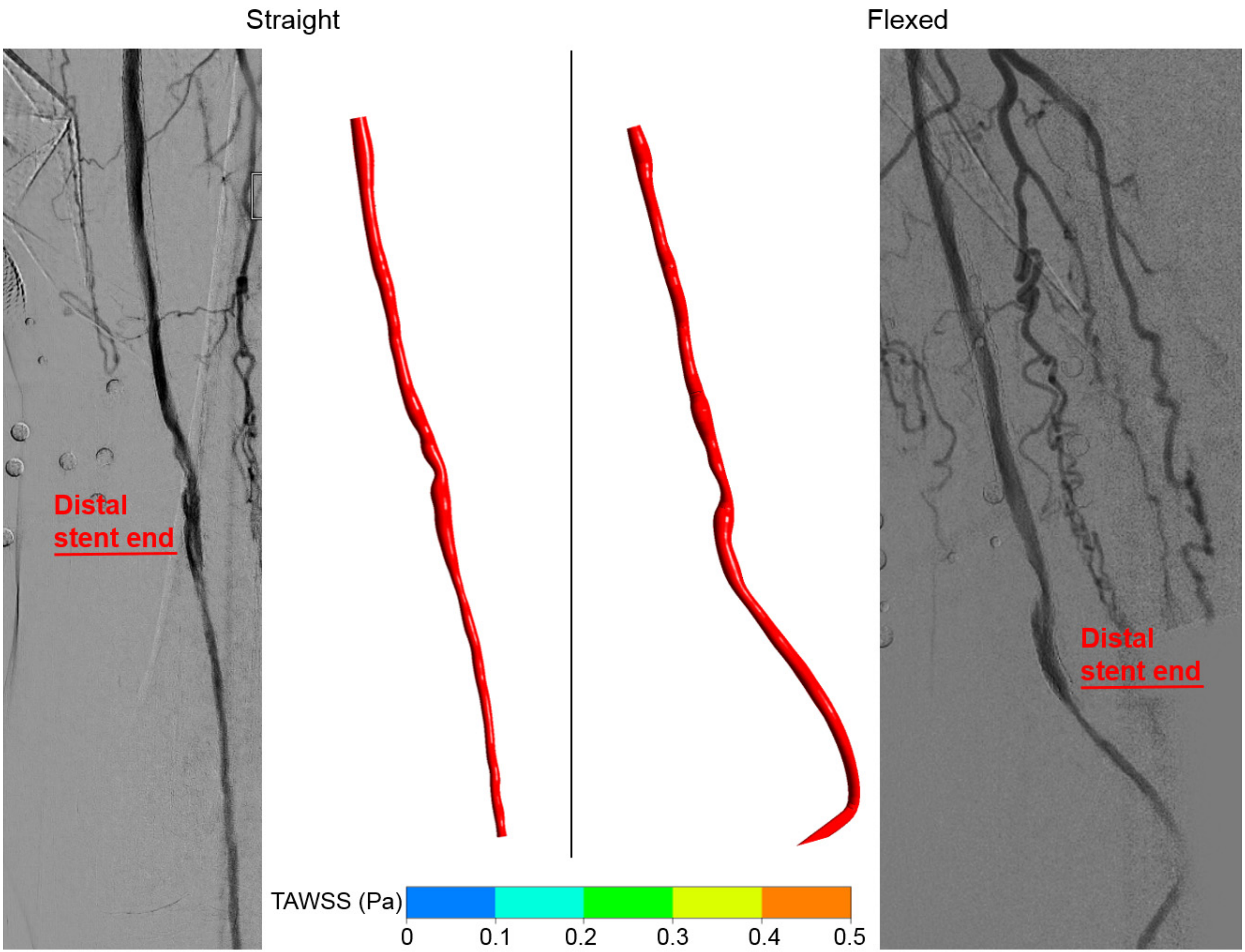

Patient 9 – Stent

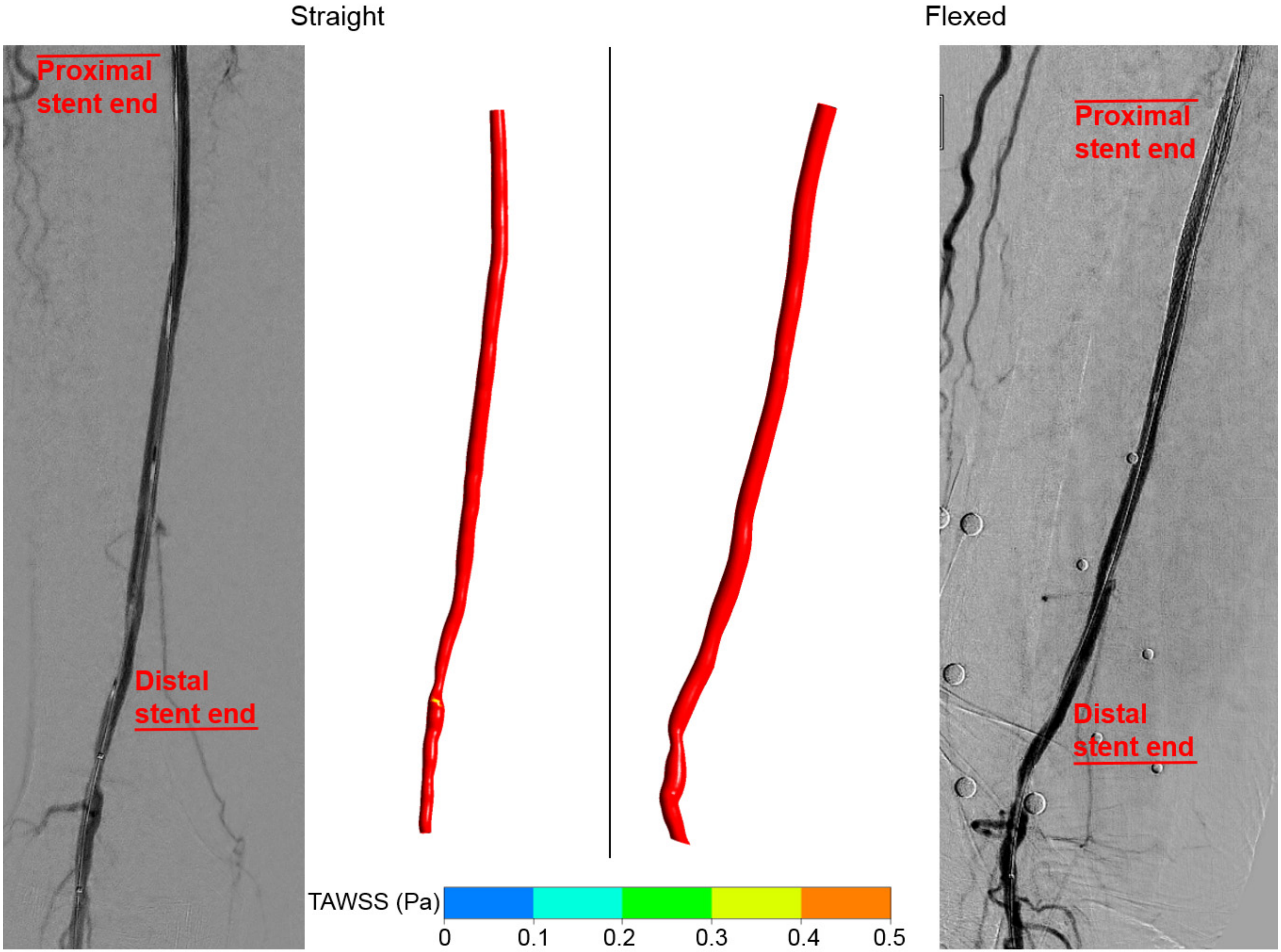

Patient 10 – Stent

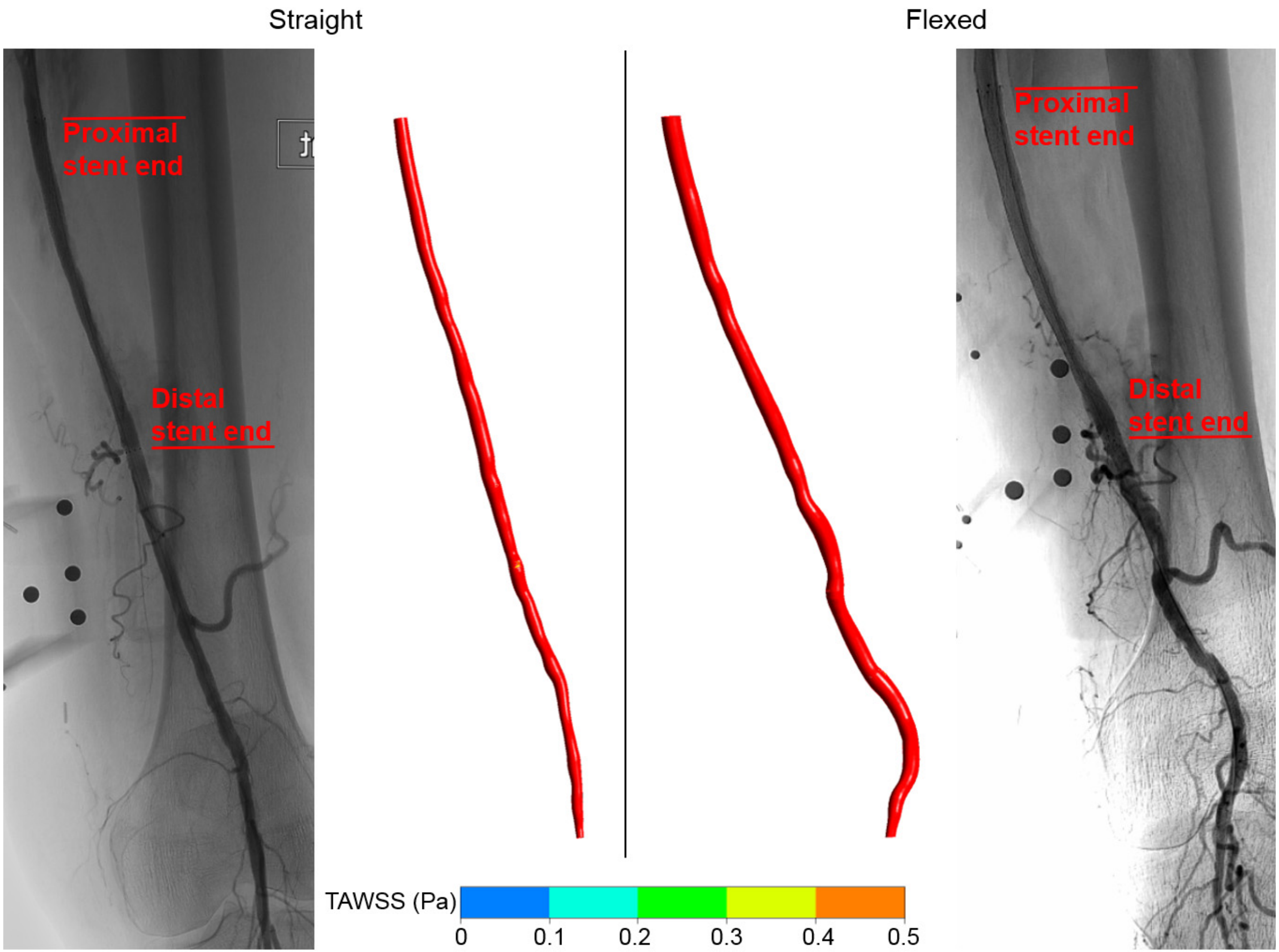

Patient 11 – PTA / Restenosis

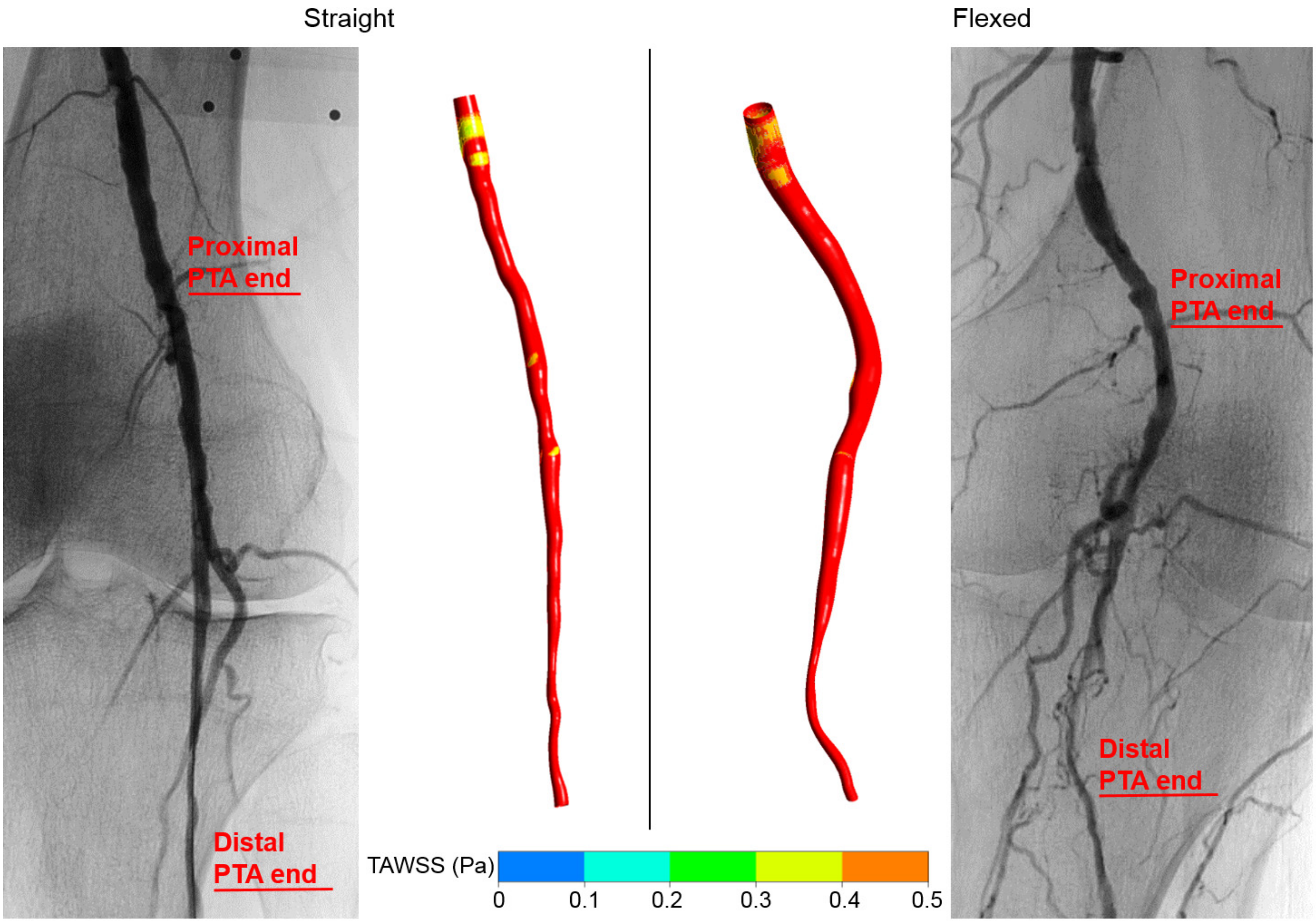

Patient 12 – PTA / Restenosis

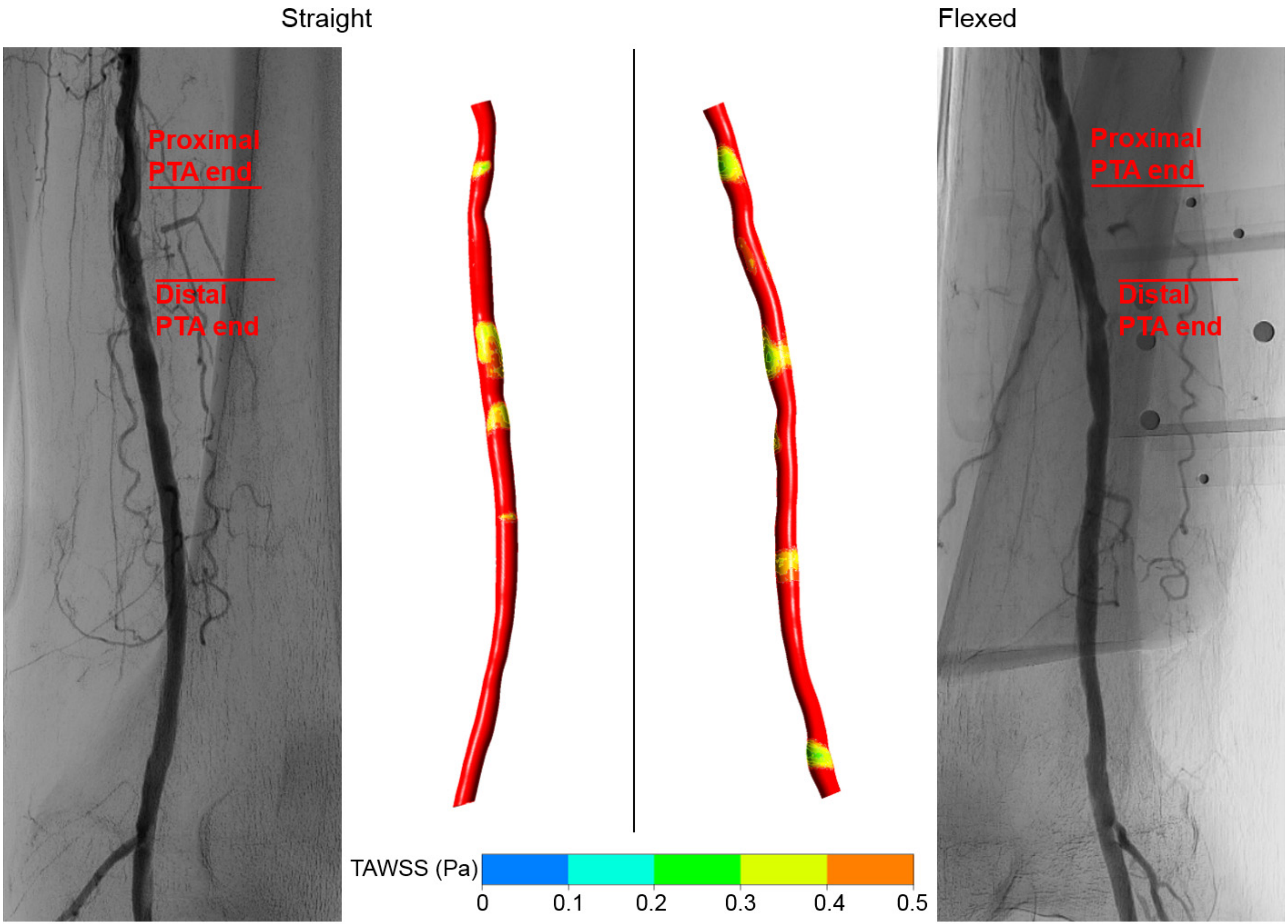

Patient 13 – PTA

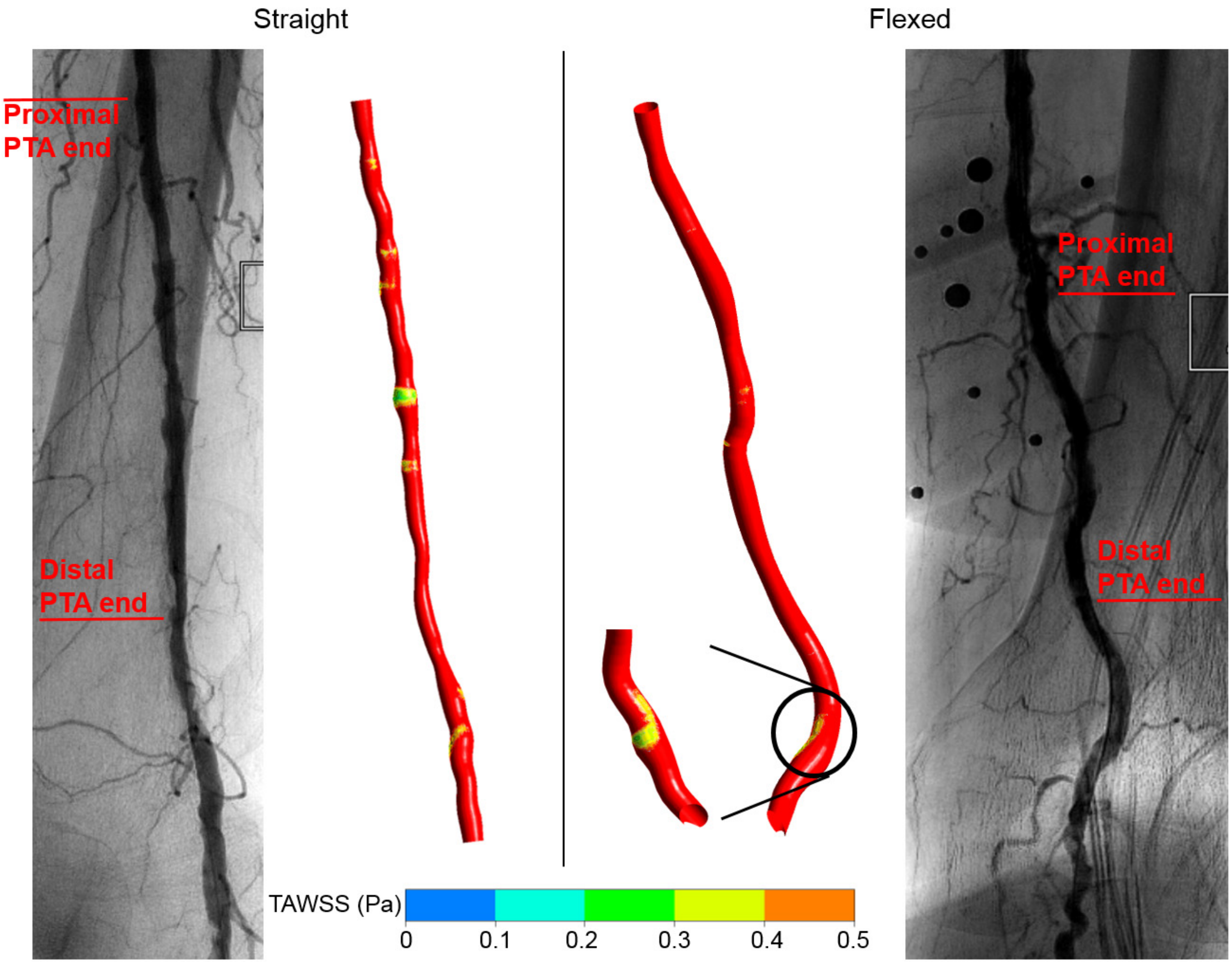

Patient 14 – PTA

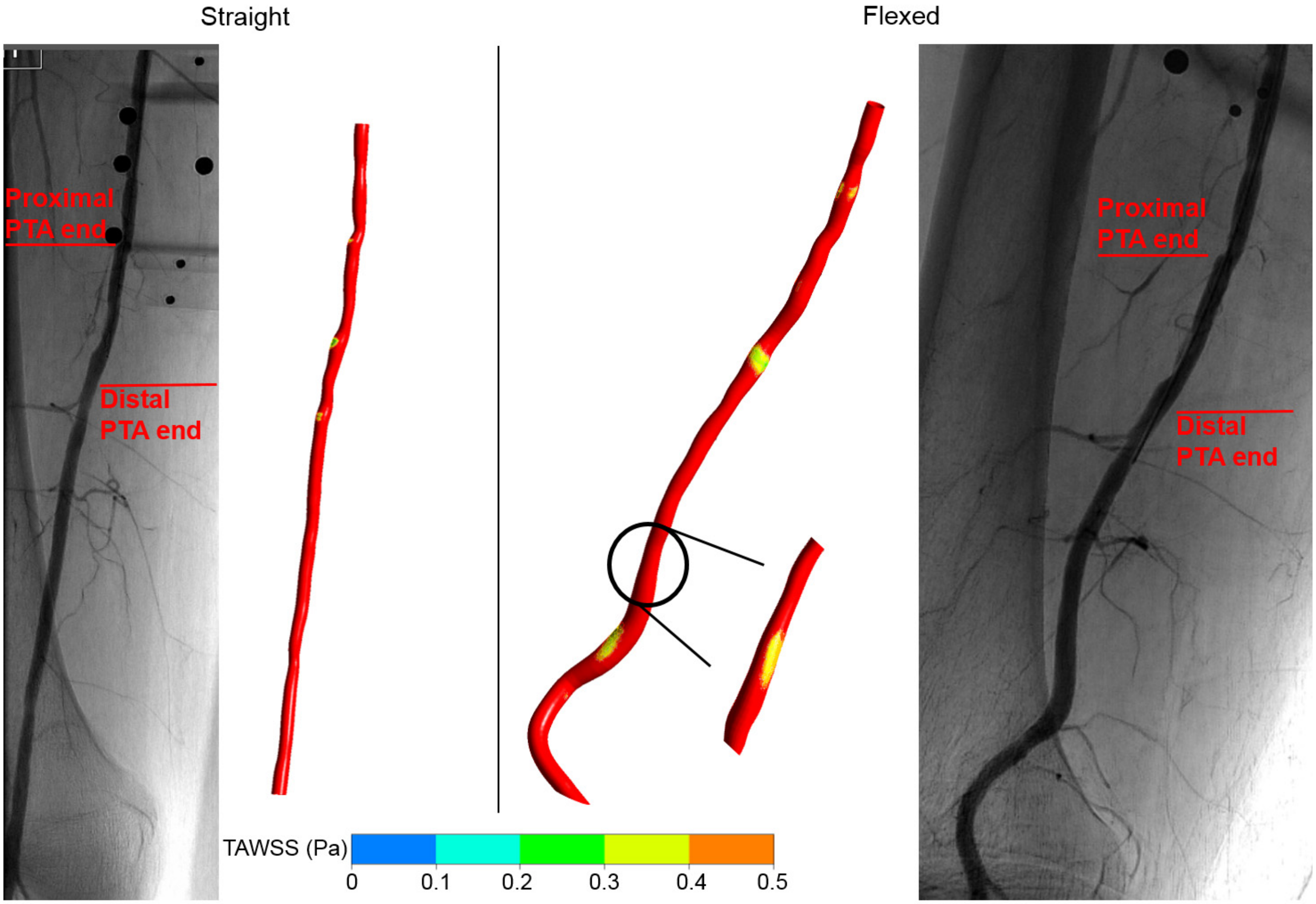

Patient 15 – PTA

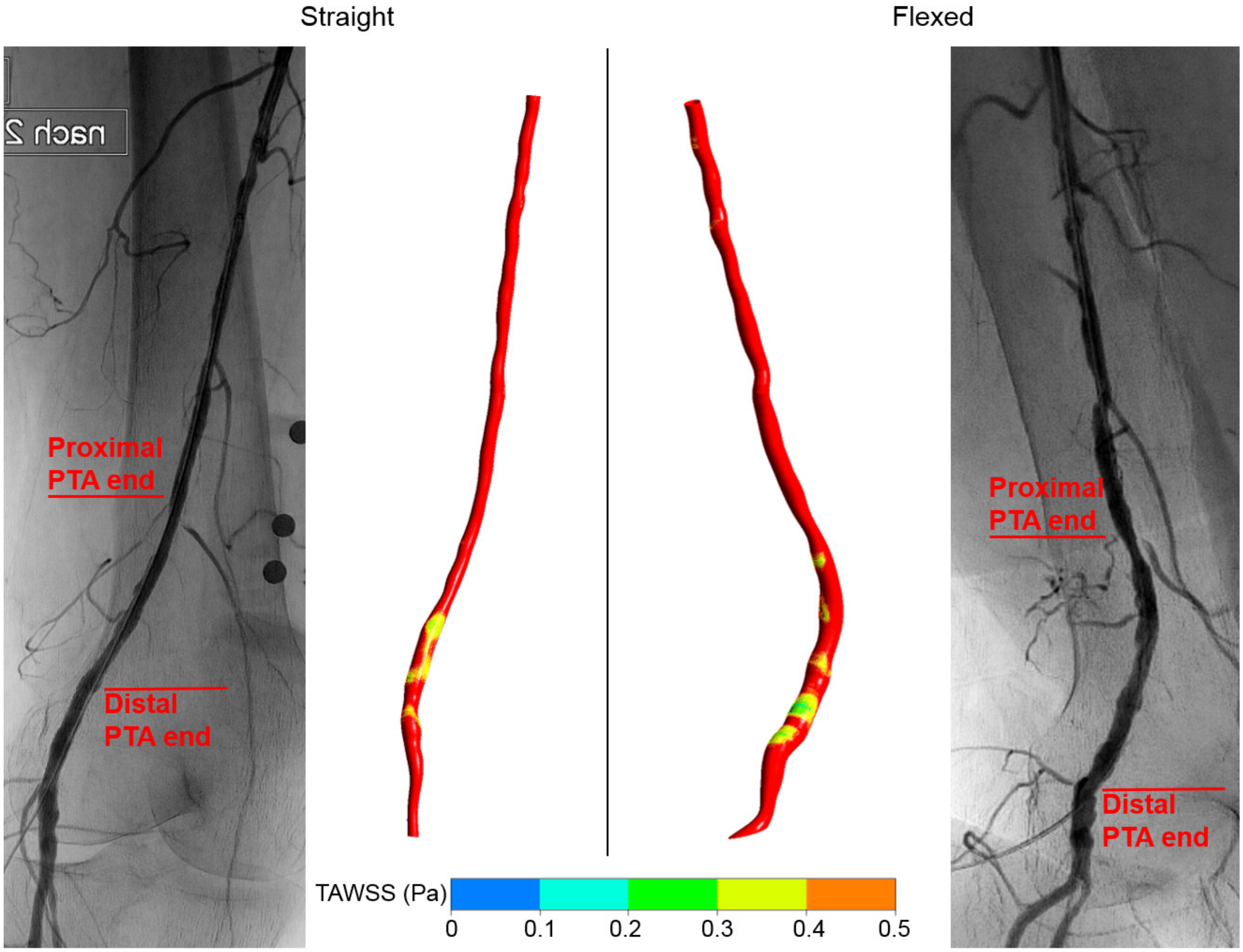

Patient 16 – PTA

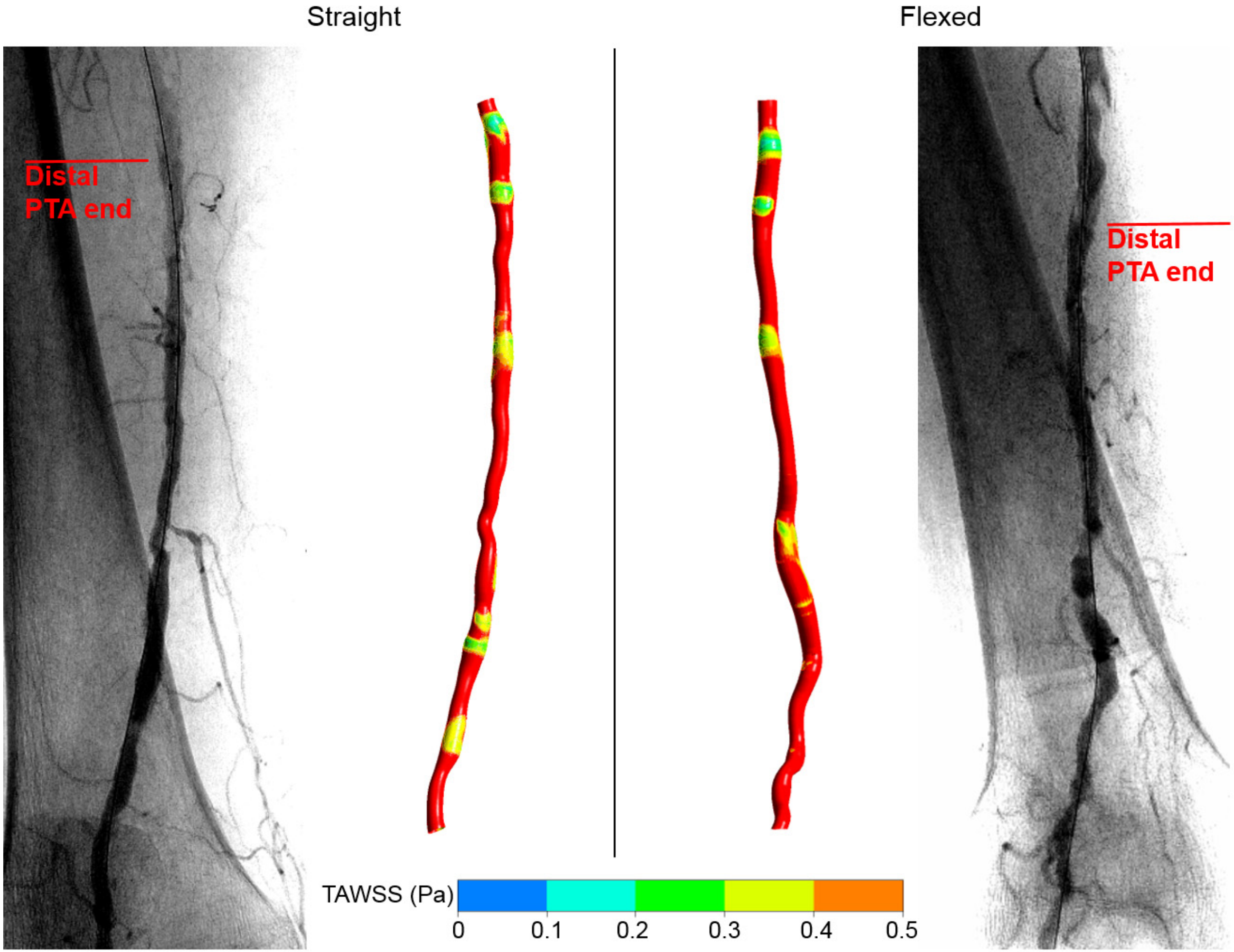

Patient 17 – PTA

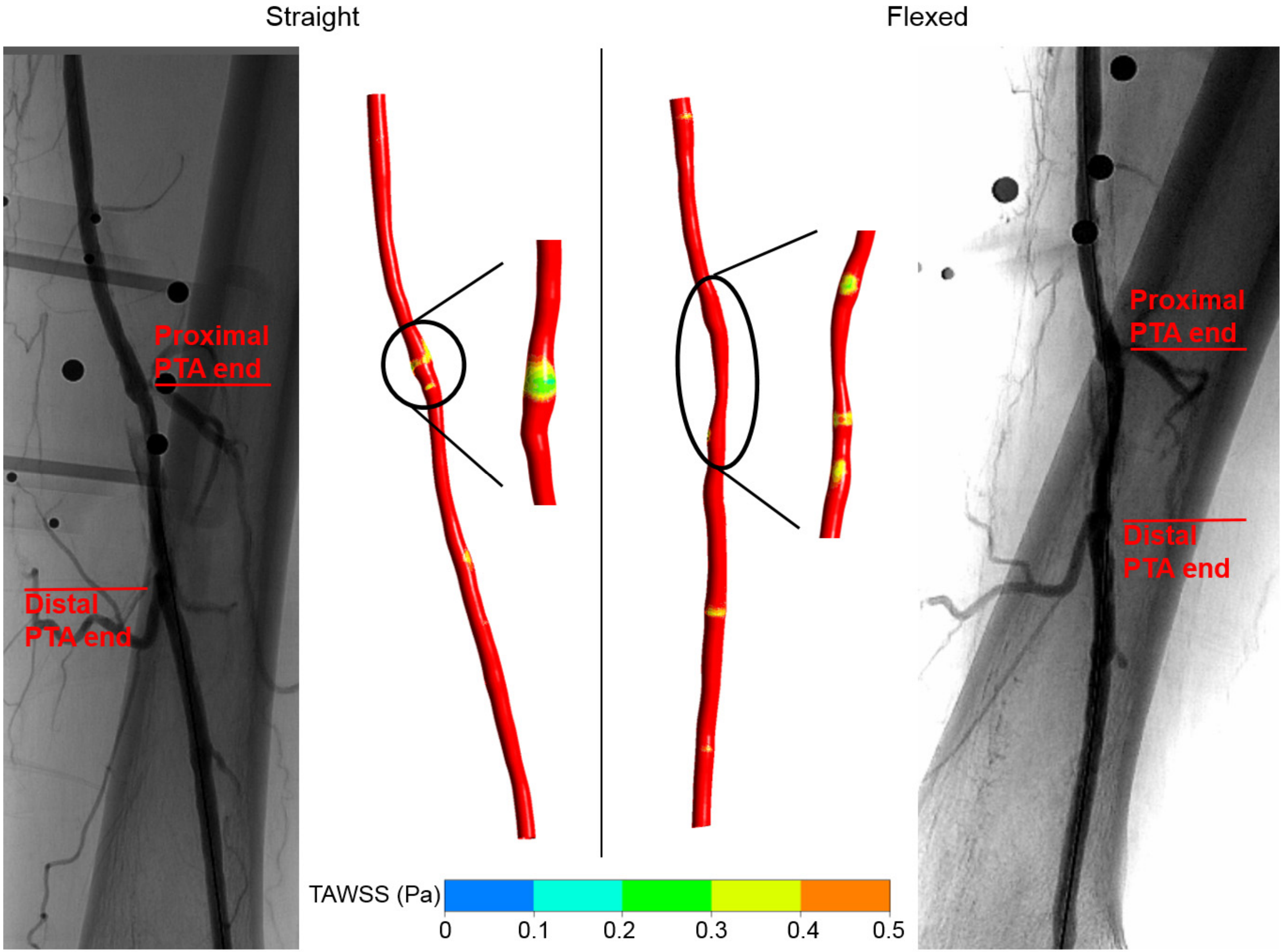

Patient 18 – PTA

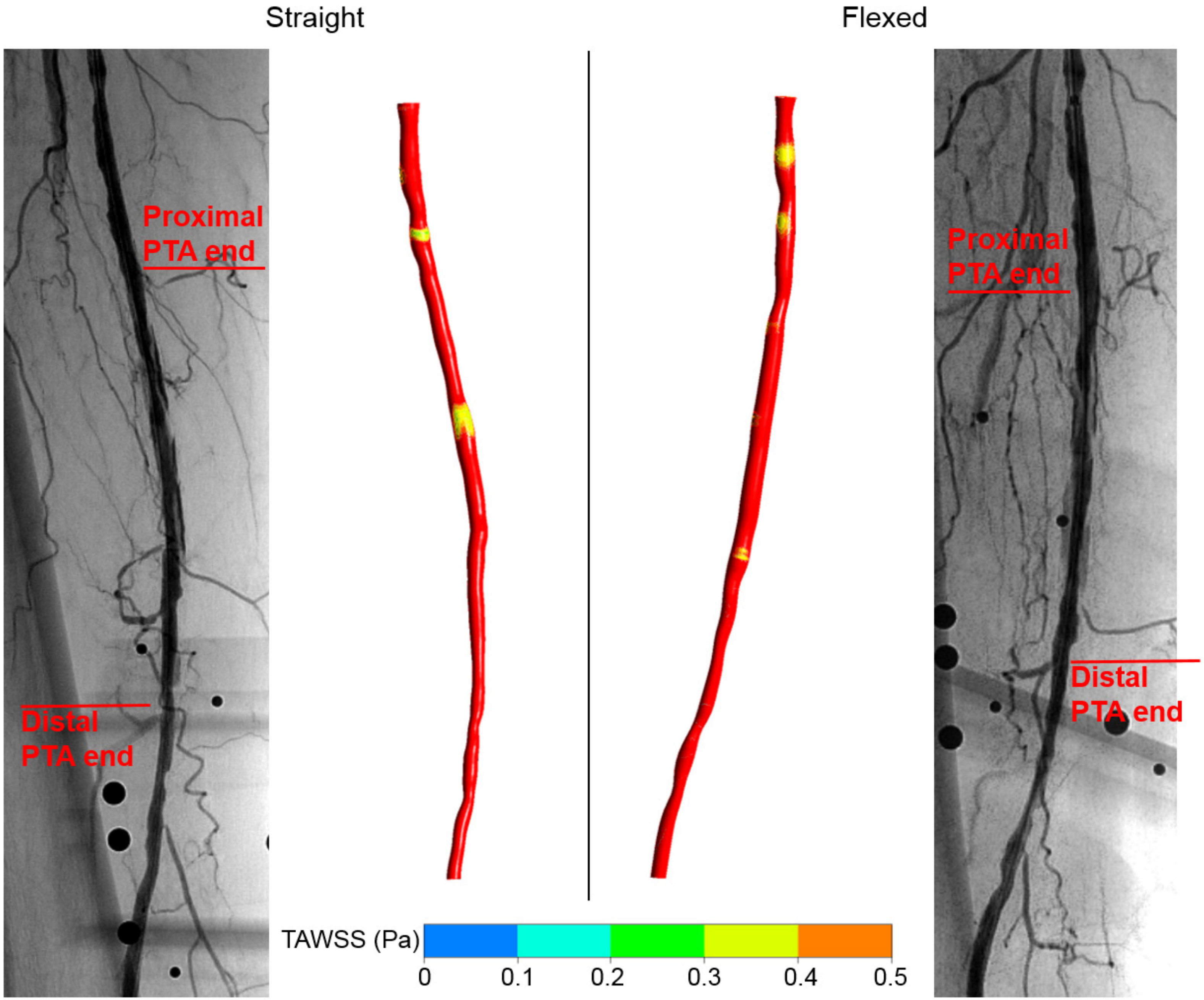

Patient 19 – PTA

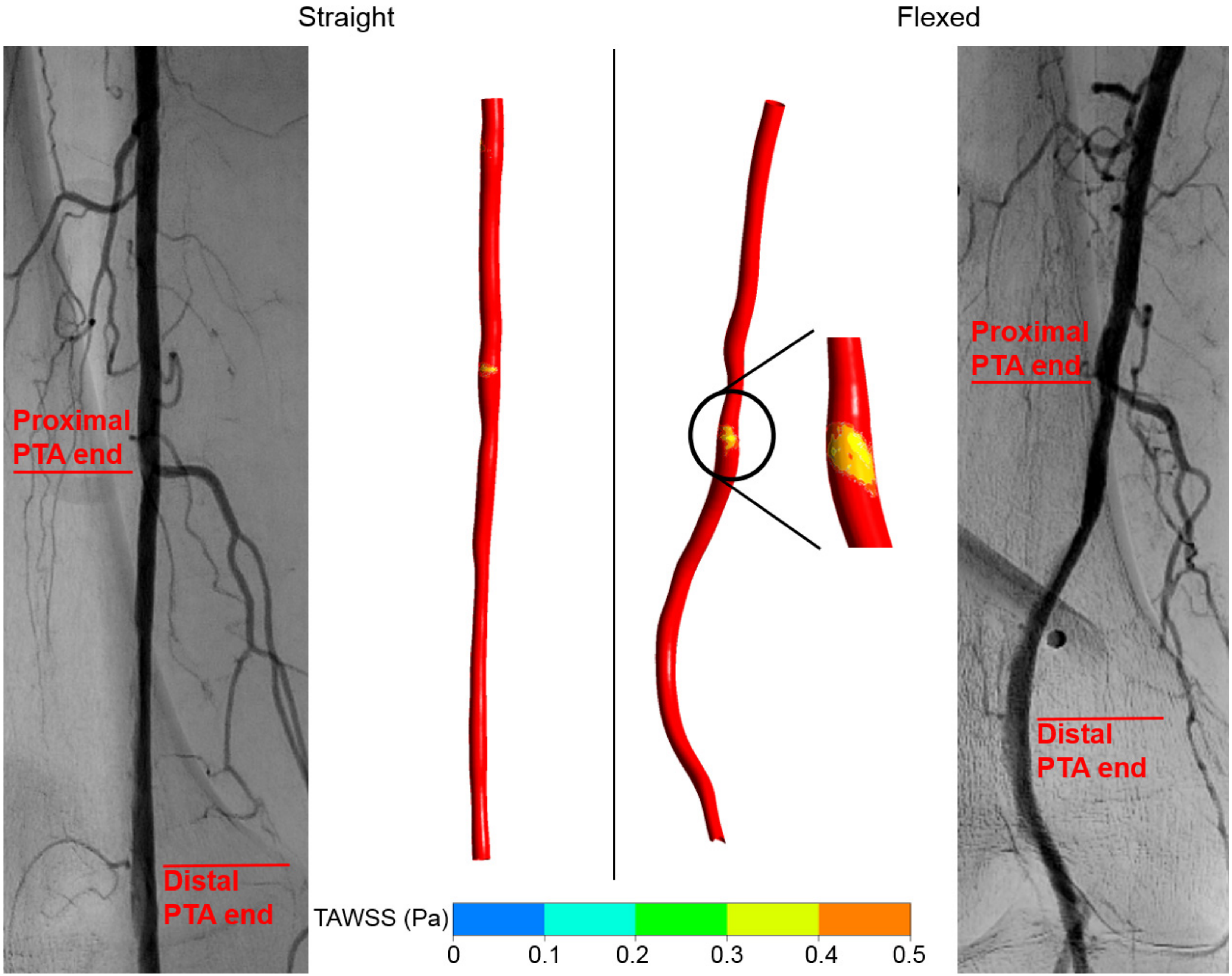

Patient 20 – PTA

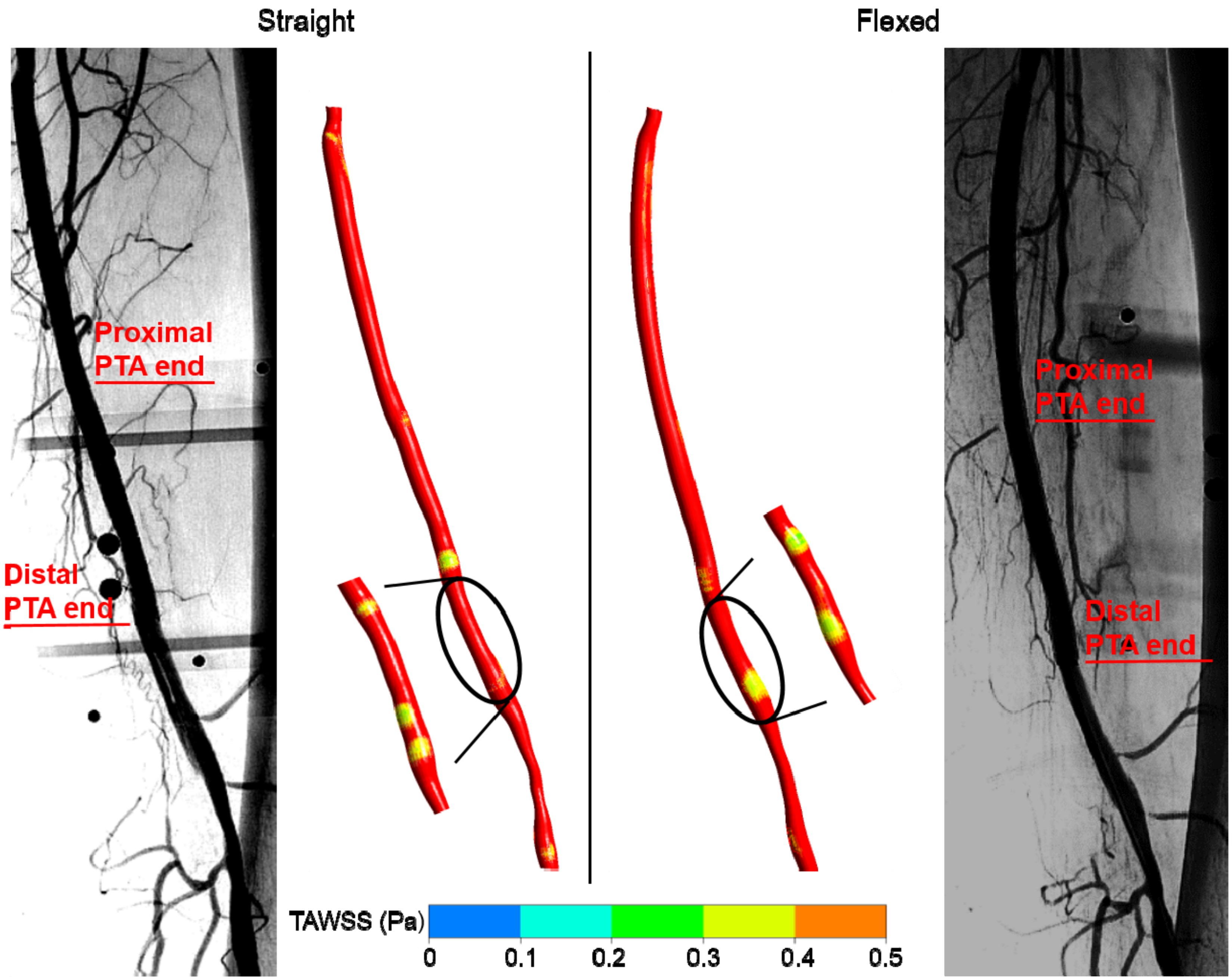

Supplement: Supplementary file 2 — Supplementary material 2 (PDF 6652 kb) [file 10237_2019_1183_MOESM2_ESM.pdf]
